# Calculation of accurate interatomic contact surface areas for the quantitative analysis of non-bonded molecular interactions

**DOI:** 10.1093/bioinformatics/btz062

**Published:** 2019-01-30

**Authors:** Judemir Ribeiro, Carlos Ríos-Vera, Francisco Melo, Andreas Schüller

**Affiliations:** Department of Molecular Genetics and Microbiology, School of Biological Sciences, Pontificia Universidad Católica de Chile, Santiago, Chile

## Abstract

**Summary:**

Intra- and intermolecular contact surfaces are routinely calculated for a large array of applications in bioinformatics but are typically approximated from differential solvent accessible surface area calculations and not calculated directly. These approximations do not properly take the effects of neighboring atoms into account and tend to deviate considerably from the true contact surface. We implemented an extension of the original Shrake-Rupley algorithm to accurately estimate interatomic contact surface areas of molecular structures and complexes. Our extended algorithm is able to calculate the contact area of an atom to all nearby atoms by directly calculating overlapping surface patches, taking into account the possible shielding effects of neighboring atoms. Here, we present a versatile software tool and web server for the calculation of contact surface areas, as well as buried surface areas and solvent accessible surface areas (SASA) for different types of biomolecules, such as proteins, nucleic acids and small organic molecules. Detailed results are provided in tab-separated values format for analysis and Protein Databank files for visualization. Direct contact surface area calculation resulted in improved accuracy in a benchmark with a non-redundant set of 245 protein–DNA complexes. SASA-based approximations underestimated protein–DNA contact surfaces on average by 40%. This software tool may be useful for surface-based intra- and intermolecular interaction analyses and scoring function development.

**Availability and implementation:**

A web server, stand-alone binaries for Linux, MacOS and Windows and C++ source code are freely available from http://schuellerlab.org/dr_sasa/.

**Supplementary information:**

Supplementary data are available at *Bioinformatics* online.

## 1 Introduction

Molecular interactions are a fundamental principle of all biological processes. Non-bonded interactions between the binding partners of a molecular complex may be modeled by considering the complementarity of the shape and physicochemical properties of the binding interface. A common way to measure shape complementarity is the estimation of the buried surface area of a molecular complex. Surface area-based methods have been employed successfully in various areas of bioinformatics including the estimation of the binding free energy (Genheden and Ryde, 2015), scoring functions for molecular docking and drug design (Sobolev *et al.*, 1996), protein structure modeling (Sobolev and Edelman, 1995), statistical potentials (McConkey *et al.*, 2003) and prediction of non-covalent contacts (Sobolev *et al.*, 1999).

Interatomic contact surface areas (CSA) may be estimated approximately by calculating differences in solvent accessible surface areas (SASA) of artificially rearranged molecular objects (Ribeiro *et al.*, 2015; Sagendorf *et al.*, 2017). However, these approximations are not very accurate and produced an average relative difference of −40% (see below).

Here, we present a new software tool dubbed *dr_sasa* for the direct calculation of interatomic CSA by a modified Shrake-Rupley algorithm (Shrake and Rupley, 1973). Our extended algorithm is able to calculate the contact area of an atom to all nearby atoms by directly calculating overlapping surface patches, taking into account the possible shielding effects of neighboring atoms. To our knowledge, a free, open-source software tool and web server for the calculation of interatomic contact surfaces between atoms of different types of biomolecules (proteins, nucleic acids and small molecules) is currently not available. For convenience, *dr_sasa* is also able to calculate buried surface areas (BSA) and solvent accessible surface areas (SASA) and accepts files in either Protein Databank (.pdb) or Tripos Mol2 (.mol2) formats.

## 2 Implementation

**Fig. 1.**
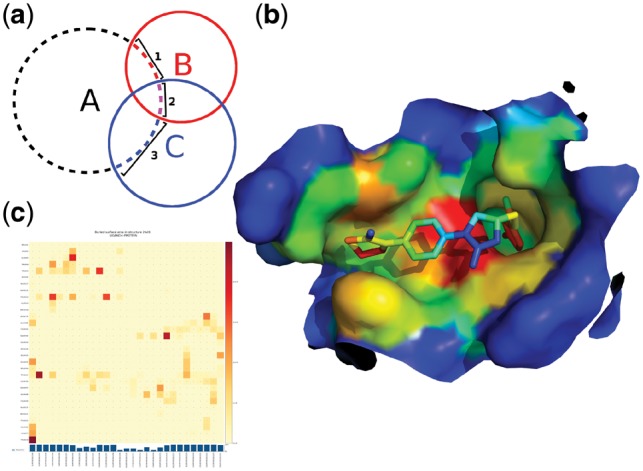
*dr_sasa* algorithm and calculation examples. **a**) 2D diagram of the calculation of contact surface areas. Letters denote atoms, circles denote the vdW surface + 1.4 Å and numbers denote contact surfaces of atom ‘A’. **b**) Surface representation of the binding site of factor Xa bound to rivaroxaban, shown as sticks (PDB ID 2w26), colored according to CSA (*dr_sasa* mode 4; blue to red: low to high Å^2^). The figure was generated with PyMOL. **c**) Surface-based contact map of factor Xa and rivaroxaban

To calculate interatomic CSA we extended the Shrake-Rupley algorithm. The original algorithm estimates SASA by considering a sphere of equidistant points for each atom, where the radius corresponds to the vdW radius of an atom plus the radius of a water molecule (1.4 Å by default). Points located inside the volume of other point clouds are excluded and SASA is estimated by counting the remaining points followed by multiplication with the surface area they represent. In our modification, instead of excluding points, which are inside the point clouds of other atoms, we store the identity of these interacting atoms. It should be noted that the surface points of an atom may be included in the volumes of multiple other atoms. For all buried surface points of an atom we therefore find all unique groups of interacting atoms. The final contact surface between two atoms is calculated by adding up the surface areas corresponding to all interacting groups of the first atom, which contain the second atom. In order to avoid overestimation of the contact surface by counting the surface area shared by a group of atoms several times, the algorithm divides this surface area by the number of atoms in the group. In Figure 1a the contact surface of atom A with atom B is calculated as the sum of surface 1 and half of surface 2, since surface 2 is shared between atoms B and C (c.f. Supplementary Section 1 for more details and pseudo code). Surface area calculations depend on the employed atomic vdW radii. *dr_sasa* uses two sets of vdW radii definitions by default: (i) for Protein Databank files the definitions as published by Chothia, 1975 are employed, which are equivalent to the radii of the popular software tool NACCESS (Hubbard and Thornton, 1993); (ii) for files provided in the Tripos Mol2 format vdW radii according to Tsai *et al.* (1999) are utilized, based on a more fine-grained atom typing scheme and similar to the molecular modeling software UCSF Chimera (Pettersen *et al.*, 2004).

In a benchmark with a non-redundant set of 245 protein–DNA complexes derived from the Protein–DNA Interface Database (PDIdb) (Norambuena and Melo, 2010) we determined that direct calculation of CSA by *dr_sasa* is more accurate and that SASA-based approximations (Ribeiro *et al.*, 2015; Sagendorf *et al.*, 2017) underestimated protein–DNA contact surfaces on average by 40% (Supplementary Section 2.1). We further validated SASA calculations with a non-redundant set of 290 protein-ligand complexes derived from PDBbind (Liu *et al.*, 2017). We compared *dr_sasa* against NACCESS, FreeSASA (Mitternacht, 2016) and MSMS (Sanner *et al.*, 1996), and obtained a low relative difference <1% (Supplementary Section 2.3). The reader is referred to the Supporting Information for extensive benchmark results and further analyses. *dr_sasa* supports four modes of operation. The fallback mode of operation (mode 0) calculates only the SASA of an input structure and outputs a text file with tabulated data and a PDB file, where SASA is stored in the B-factor column. In the contact surface area (CSA) mode of operation (mode 1) the program calculates a complete matrix of all surface-based contacts per atom and per residue and saves them as tab-separated values files. The sum over columns in these matrices is equivalent to the overall BSA of an atom or residue. Theses sums are also saved in the B-factor column of a separate PDB output file for visualization (Fig. 1b). In addition, contact map images may be generated from these matrices with a separate Python script (Fig. 1c). The CSA-mode requires the selection of chains to be considered as separate objects, or in case the input file contains different types of biomolecules (protein and nucleic acids, protein and ligands, or any combination of these) the program can automatically identify the separate objects and calculate their interactions. Modes of operation 2 and 3 calculate intramolecular surface-based contact maps per residue and per atom. The last mode of operation (mode 4) calculates intermolecular CSA without requiring that the contact surfaces are solvent accessible. This is especially useful for internal and deep ligand binding cavities (Fig. 1b).

*dr_sasa* is suitable for batch processing. SASA calculations for 10 000 PDB snapshots of a molecular dynamics trajectory of a protein–DNA complex (1830 atoms) took 36 min. on a 16-thread x86 notebook computer (AMD Ryzen 7 1700 @ 3.2 GHz), equivalent to 0.2 s per structure. CSA calculations on the same dataset took 214 min. (1.3 s per structure).

In conclusion, we present a freely available, versatile command-line program and web server for the accurate calculation of interatomic contact surfaces by a modified Shrake-Rupley algorithm, which may be useful for the quantitative analysis of non-bonded molecular interactions.

## Supplementary Material

btz062_Supplementary_Information
