# Supplementary material for: Calculation of accurate interatomic contact surface areas for the quantitative analysis of non-bonded molecular interactions
Source: Bioinformatics. 2019 Jan 30;35(18):3499–501. doi: 10.1093/bioinformatics/btz062 (PMC6748739; doi:10.1093/bioinformatics/btz062)
Supplement: btz062_Supplementary_Information [file btz062_supplementary_information.pdf]

Supplementary Information

Calculation of accurate interatomic contact surface areas for the quantitative analysis of non-bonded molecular interactions

Judemir Ribeiro[1], Carlos Ríos-Vera[1], Francisco Melo[1]* and Andreas Schüller[1]*

[1]Departamento de Genética Molecular y Microbiología, Facultad de Ciencias Biológicas, Pontificia Universidad Católica de Chile, Alameda 340, Santiago, Chile.

*To whom correspondence should be addressed: aschueller@bio.puc.cl ; fmelo@bio.puc.cl

Availability and implementation: A web server, stand-alone binaries for Linux, MacOS and Windows, and C++ source code are freely available from http://schuellerlab.org/dr_sasa/.

Table of Contents

1 Methods *2*

1.1 Calculation of the solvent accessible surface area of a molecule 2

1.2 Calculation of contact surface areas 2

1.3 Definitions of van der Waals radii 4

1.4 Program usage 6

1.5 Generation of graphical contact map plots 6

1.6 Web server 6

1.7 Output files 7

1.8 Feature comparison 8

1.9 Protein-DNA validation dataset 9

1.10 Protein-ligand validation dataset 9

1.11 Runtime comparison 9

2 Benchmarks *10*

2.1 Improved accuracy of contact surface area calculations by our modified Shrake-Rupley algorithm 10

2.2 Validation of buried surface area calculations 14

2.3 Validation of solvent accessible surface area calculations for protein-ligand complexes 17

2.4 Validation of solvent accessible surface area calculations for protein-DNA complexes 21

2.5 Effect of different vdW radii definitions on SASA calculation 23

2.6 Calculation of CSA without requiring that the contact surfaces are solvent accessible 26

3 Acknowledgements *28*

4 References *29*

1 Methods

1.1 Calculation of the solvent accessible surface area of a molecule

For the calculation of the solvent accessible surface area or SASA, the Shrake and Rupley algorithm was used (Shrake and Rupley, 1973). It consists in generating a spherical cloud of points for each atom, where each point is at a distance equivalent to the van der Waals (vdW) radius plus the radius of a water molecule (1.4 Å by default) from the atom center. Each point represents a surface segment whose size is equivalent to the total surface divided by the amount of points. After excluding the points that are located inside the volume of the point clouds of other atoms, it is possible to obtain the accessible surface area by counting the remaining points and multiplying by the surface area they represent. The point cloud must have distances between points as equivalent as possible in the spherical plane to remove inaccuracies in the surface calculation caused by the uneven distribution of points. This was solved by approximating the surface of a sphere by the tessellation algorithm implemented in the software Thomson Applet (Saff and Kuijlaars, 1997; Cecka *et al.*, 2007) (Figure S1). Briefly, the algorithm initially generates points on a sphere at random positions. Then, a gradient descent algorithm that simulates each point as an electrical charge constrained to move on the spherical surface is executed to subsequently optimize the position of these points. The optimization ends when the variance of the distance between points no longer decreases. We precalculated point clouds of a unit sphere with 15,092 points.

To optimize the search for interacting atoms, only those atoms that have their centers at a distance less than two times the sum of their van der Waals radii plus two times the van der Waals radius of a water molecule (1.4 Å) are considered as potentially interacting.

1.2 Calculation of contact surface areas

To calculate the contact surfaces between two atoms, we extended the original Shrake-Rupley algorithm (Shrake and Rupley, 1973). In our modification, new variables were introduced to store the additional information of which atoms bury a certain surface point of another atom (Figure S2). With this information it is possible to find groups of points of an atom that are buried by other unique groups of atoms, identifying all unique groups. Our algorithmic modifications include the structures *pBuriedBy* and *AreaBuriedBy* that allow to store the information of which points in the surface of an atom are buried by which other groups of atoms. The latter variable contains the list of unique set of atoms and the number of points that each of them buries (Figure S2). When applied to the simplest example of a three-body system, the algorithm proceeds as follows (Figure S3): The surface of body 1 buried by body 2 corresponds to the section in red, plus the purple section. Since the purple area was counted twice, it is divided by two for each atom. In general, the value of the buried surface area that a body B causes to a body A is equivalent to the sum of all overlaps divided by the number of atoms that participate with the group of body B. In the example of Figure S3, the contact surface area of body 1 that is buried by body 2 is equivalent to the section in red plus half of the purple section. This normalization of shared contact surfaces by the number of interacting atoms ensures that the sum of all contact surfaces is equal to the buried surface area calculated by the original Shrake-Rupley algorithm.

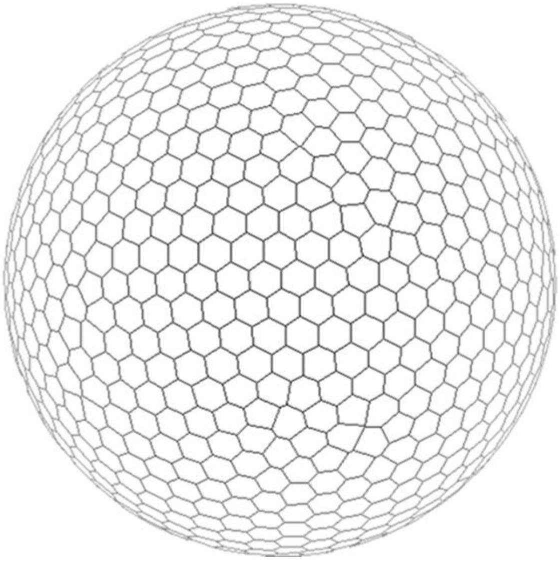

Figure S1. Sphere surface approximation calculated by the Thomson Applet software. In this example a sphere was subdivided in 1,000 equivalent areas. Each subsurface is represented by a center point (not shown). In practice, *dr_sasa* works with a precalculated unit sphere with 15,092 points.

Algorithm 2 Steps to calculate interaction sub surfaces

```
procedure CalculateDBSA
    #Structure PDB
    pdbstrudct pdb
    #3xN matrix with the 3D coordinates of the point cloud in a unit sphere
    matrix2D unitsphere
    for atomI in pdbstruct do
        #Stores the lists of atoms that bury a certain point in the surface of atom I
        map pBuriedBy
        #Stores the unique sets of atoms found and the amount of points that they bury
        list AreaBuriedBy
        #Scales and moves a copy of the point cloud to the center of the current atom
        matrix2D points ← unitsphere ∗ (atomI.vdw + 1.4) + atomI.coords
        #Iterates of all atoms J that interact with atom I
        for atomJ in atomI.interactions do
            for point in points do
                #Checks if point is inside the volume of atom J
                if IsPointInVolume(atomJ, point) then
                    #Adds atom to the list of atoms that bury this point
                    pBuriedBy[point].add(atomJ)
        #Process the sets and calculates the buried area caused by each one
        AreaBuriedBy ← ParseSets(pBuriedBy)
        #Stores the interactions in the atom object
        atomI.AreaBuriedBy ← AreaBuriedBy
```

Figure S2. Our extension of the original Shrake-Rupley algorithm to calculate contact surface areas between atoms.

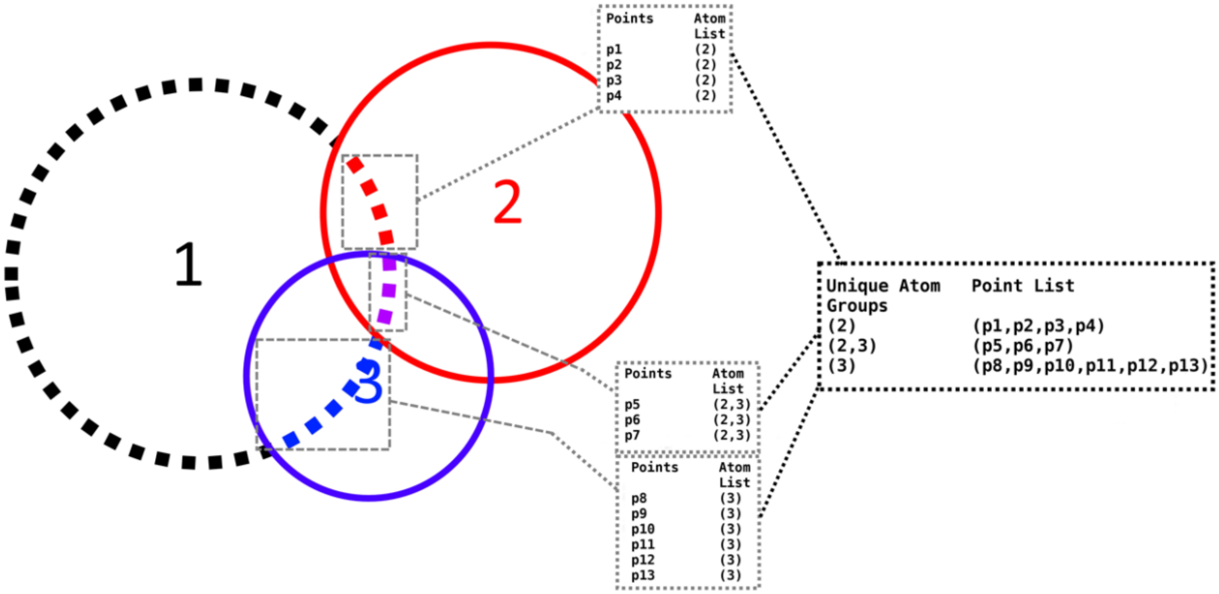

Figure S3. 2D diagram of the algorithm for the calculation of contact surface areas. In this three-body example, the body 1 has its surface buried by bodies 2 and 3. Each point on surface of 1 represents a value equivalent to (total surface)/(number of points). The points in red represent the surface buried only by body 2, the blue points are buried only by 3, and the purple points are buried by bodies 2 and 3. In the outlined boxes in the center part of the figure, representations of maps of buried points to list of atoms are shown, which corresponds to variable *pBuriedBy* in the algorithm described in Fig S2. The outlined box on the right-hand side corresponds to the variable *AreaBuriedBy* of our algorithm and maps unique groups of atoms to lists of surface points, which correspond to contact surface areas.

1.3 Definitions of van der Waals radii

Structures provided in PDB and Mol2 formats use different definitions of vdW radii. For PDB files the vdW radii definition is equivalent to the radii of the popular software tool NACCESS (Hubbard and Thornton, 1993; Chothia, 1975) and is provided in Table S1 and Table **S2**. Structures provided in the Mol2 format are assigned vdW radii according to the SYBYL atom types contained in Mol2 files. This vdW radii definition is equivalent to the one used by the molecular modeling program UCSF Chimera (Pettersen *et al.*, 2004) based on the data of Tsai *et al.*, 1999 (Table S3). vdW radii of ions were extracted from the CRC Handbook of Chemistry and Physics (Lide, 2001) for both PDB and Mol2 files, as in UCSF Chimera (Pettersen *et al.*, 2004) (Table S4). Ions are assumed to be in their prevalent coordination number. Hydrogen atoms are ignored in all calculations. User-defined tables of vdW radii may be provided as plain text files as exemplified in the online documentation of *dr_sasa* (command line switch: -v).

Table S1. Van der Waals radii used in PDB file format for proteins.

Element	Name list	Radius (Å)	Polarity
C	All CA, all CB, PQLMRKE-CG, ML-CD, IL-CD1, L-CD2, VL-CG1, TVL-CG2	1.87	Hydrophobic
C	All C, FYWH-CD2, CZ2, CZ3, NFYWHD-CG, QE-CD, FYR-CZ, CH2, CE3, FYW-CD1, FYH-CE1, FYW-CE2	1.76	Hydrophobic
O	All oxygens	1.40	Polar
N	All nitrogens except lysine NZ	1.65	Polar
N	Lysine NZ	1.50	Polar
S	Methionine and cysteine sulfurs	1.85	Hydrophobic

Atoms not listed in the table are assigned a value based on their chemical element. These are (in Å) 1.8, 1.6, 1.4, 1.9, 1.85, 1.9 for C, N, O ,P ,S, Se, respectively, and 2.094, 1.560, 1.978, 1.735 Å for I, F, Br, Cl, respectively (taken from Table S3). The name list includes single letter amino acid codes and PDB atom ids. Atom types are classified into 'hydrophobic' and 'polar' according to the polarity of their typical bonds formed in organic molecules.

Table S2. Van der Waals radii used in PDB file format for nucleic acids.

Element	Name list	Radius (Å)	Polarity
C	All carbons	1.80	Hydrophobic
O	All oxygens	1.40	Polar
P	All phosphorus	1.90	Polar
N	All nitrogen	1.60	Polar

Atoms not listed in the table are assigned a value based on their chemical element. These are (in Å) 1.8, 1.6, 1.4, 1.9, 1.85, 1.9 for C, N, O ,P ,S, Se, respectively, and 2.094, 1.560, 1.978, 1.735 Å for I, F, Br, Cl, respectively (taken from Table S3).

Table S3. Van der Waals radii used for Mol2 files.

Element	SYBYL atom type	Radius (Å)	Polarity
C	C.3 C.1 C.2	1.88	Hydrophobic
C	C.cat	1.88	Polar
C	C.2	1.76	Hydrophobic
C	C.ar	1.61	Hydrophobic
N	N.4 N.3 N.2 N.pl3 N.1 N.am N.ar	1.64	Polar
O	O.3 O.co2	1.46	Polar
O	O.2 O	1.42	Polar
S	S.3 S.2	1.77	Hydrophobic
S	S.O2 S.O	1.77	Polar
P	P.3	1.871	Polar
Cl	CL	1.735	Hydrophobic
F	F	1.560	Hydrophobic
Br	BR	1.978	Hydrophobic
I	I	2.094	Hydrophobic

The radii are equivalent to the definition of UCSF Chimera (Pettersen *et al.*, 2004) based on Tsai *et al.*, 1999.

Table S4. Van de Waals radii for common ions.

Ion	Radius (Å)	Ion	Radius (Å)	Ion	Radius (Å)
Al^{+3}	0.54	Ga^{+3}	0.62	Pt^{+2}	0.80
As^{+3}	0.58	Ge^{+2}	0.73	Rb^{+}	1.52
Au^{+1}	1.37	Hg^{+2}	1.02	Sb^{+3}	0.76
Ba^{+2}	1.35	K^{+}	1.38	Sc^{+3}	0.75
Be^{+2}	0.45	Li^{+}	0.76	Sn^{+4}	0.69
Bi^{+3}	1.03	Mg^{+2}	0.72	Sr^{+2}	1.18
Ca^{+2}	1.00	Mn^{+2}	0.83	Tc^{+4}	0.65
Cd^{+2}	0.95	Mo^{+3}	0.69	Ti^{+2}	0.86
Co^{+2}	0.65	Na^{+}	1.02	V^{+2}	0.79
Cr^{+2}	0.73	Ni^{+2}	0.69	Zn^{+2}	0.74
Cs^{+}	1.67	Pb^{+2}	1.19	Zr^{+4}	0.72
Fe^{+2}	0.61	Pd^{+2}	0.86		

Common ions use the radius for their most common coordination number. The radii were extracted from CRC Handbook of Chemistry and Physics (Lide, 2001), as in UCSF Chimera (Pettersen *et al.*, 2004). All ions are classified as polar.

1.4 Program usage

This program receives inputs, sets its operation mode through the command line, and outputs its results as text files. The output tables are separated by tabs, facilitating the import of the data to most common spreadsheet software or easy parsing with custom user scripts. The standard mode of operation or operation mode 0 (command line switch: -m 0) calculates only the Solvent Accessible Surface Area (SASA) of the input molecules, where the output is a PDB file with the SASA of the atom (in Å^2) inserted in the B-factor column. The contact surface area mode of operation (mode 1, command line switch: -m 1) requires the selection of the chains to be considered as separate objects, or if the PDB or Mol2 file contains different types of biomolecules (protein and nucleic acids, protein and ligands, or any combination of the three) the program will automatically identify the separate objects and calculate their interactions. In this mode the output is presented as two types of matrices. The first type is a NxN matrix where N is the total number of atoms in the structure, and the sum of each column is equal to the total buried surface area of an atom. Theses sums are also saved in the B-factor column of a separate PDB output file for visualization. The second type of matrices are LxM sized, where L is the number of atoms of the first selected chain or molecule type and M is the number of atoms of the second selected chain or molecule type. These latter matrices come in pairs of files, where each value of the first matrix corresponds to the buried surface that chain A causes to B and the inverse is true for the second matrix. Output matrices for all possible pairs of defined or identified objects will be written. All these matrices are generated twice, at two levels: per atom and per residue. In addition, to generate intramolecular surface-based contact maps, all residues (command line switch: -m 2) and atoms (command line switch: -m 3) can be considered as objects. In these two operation modes 2 and 3, residues/atoms are compared all-against-all by first isolating a pair of residues/atoms and then calculating their contact surface. Modes of operation 1, 2, and 3 take only solvent exposed atoms into consideration. The last mode of operation (mode 4, command line switch: -m 4) calculates intermolecular contact surface areas without requiring that the contact surfaces are solvent accessible. This is especially useful for internal and deep ligand binding cavities with low solvent accessibility (see example from Fig. 1b in the main manuscript). The SASA per atom and BSA per atom are further classified according to the chemical nature of the corresponding atom into protein backbone/side chain, DNA backbone/base and polar/hydrophobic. These data are provided in two additional output files (.atmasa and .datmasa for SASA and BSA, respectively).

1.5 Generation of graphical contact map plots

Graphical surface-based contact map plots (Figure S4) can be generated with a python script freely available from our web site. Required inputs for the script is a **.by_atom.tsv* or **.by_res.tsv* matrix file and a corresponding *.atmasa* file (see Table S5 for a description of the output files and their content). The contact maps may be generated per residue, per atom or mixed (per residue for proteins and per atom for small ligand molecules). Each cell of the contact map corresponds to the contact surface area (CSA in Å) of the atom/residue on the X axis, caused by the atom/residue on the Y axis. The bar plot on the X axis indicates relative BSA: BSA is calculated as the column sum of CSA, which is subsequently normalized by the SASA of the atom/residue on the X axis (BSA_{rel} = BSA/SASA).

1.6 Web server

A web server to run *dr_sasa* is freely available from http://schuellerlab.org/dr_sasa/. The web server is able to run *dr_sasa* in all modes of operation described above and generates a compressed ZIP file for download, containing all output files. For user convenience, the web server does also generate contact map plots by default (Figure S4).

1.7 Output files

Table S5. Description of output files generated by *dr_sasa*.

File name	Description
<structure file name>**.asa.pdb**	PDB file containing all valid atoms. The B-factor column is replaced with the SASA value of the corresponding atom. Created in all operation modes.
<structure file name>**.dsasa.pdb**	PDB file containing all valid atoms. The B-factor column is replaced with the BSA value of the corresponding atom. Generated in operation modes 1 and 4.
<structure file name>**.atmasa**	Tab separated file containing all valid atoms. Each line contains the SASA value for an atom, categorized by position (e.g. backbone/sidechain) and polarity. Generated in all operation modes.
<structure file name>**.datmasa**	Tab separated file containing all valid atoms. Each line contains the BSA value for an atom, categorized by position (e.g. backbone/sidechain) and polarity. Generated in operation modes 1 and 4.
<structure file name>**.(obj_A)_vs_(obj_B).by_atom.tsv**<br><structure file name>**.(obj_B)_vs_(obj_A).by_atom.tsv**	Pair of matrices containing the information of the CSA that atoms of object B cause to object A and vice versa, respectively. This information is indicated in the header, with the format "(obj_A)<-(obj_B)". The arrow indicates that the matrix contains the CSA of atoms of A, which are buried by B.<br>The buried object is always placed first and corresponds to the X axis. The sum of all CSA values per column (column sums) corresponds to the BSA of the first object.<br>The objects can be either molecular types, chains, or chain combinations. Pairs for ALL possible combinations will be generated.<br>Summing all values equals the total buried surface area for the object indicated in the header. Created in operation modes 1 and 4.
<structure file name>**.(obj_A)_vs_(obj_B).by_res.tsv**<br><structure file name>**.(obj_B)_vs_(obj_A).by_res.tsv**	Same as the previous files, except that the atom results are summed per residue. Allows for easier analysis of bigger complexes. Generated in operation modes 1 and 4.
<structure file name>**.matrix.(selected chains).by_atom.tsv**<br><structure file name>**.matrix.(selected chains).by_res.tsv**	NxN files containing the same information as the matrix pairs, where N is equal to the total number of atoms in the structure or residue, respectively, within the selected chains. Summing the columns of this file is equal to the BSA of the atom in X axis in operation modes 1 and 4. Please note that this sum has no meaning for operation modes 2 and 3. Generated in all operation modes except mode 0.
<structure file name>**.overlaps**	File with details about all subsurface burial information. Deprecated, may be removed in upcoming software versions. Generated in all operation modes except mode 0. Optional for operation modes 2 and 3.

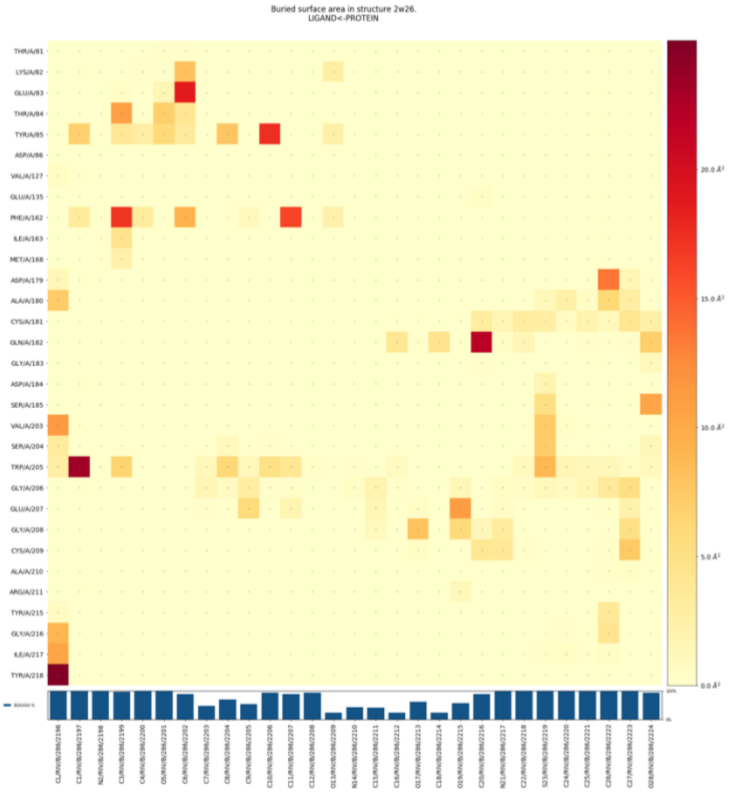

Figure S4. Surface-based contact map of a protein-ligand complex. The map represents the serine protease factor Xa bound to the small molecule inhibitor rivaroxaban (PDB ID 2w26). The figure was generated with our provided python script and it is equivalent to those images generated by the web server.

1.8 Feature comparison

Table S6 Feature comparison of different surface area software tools.

	dr_sasa	FreeSASA	NACCESS	PISA	CCP4	GetArea	DSSP	MSMS
Supports proteins	Y	Y	Y	Y	Y	Y	Y	Y
Supports DNA/RNA	Y	Y	Y	Y	Y	U		U
Supports small molecules	Y	Y	U	Y		U		U
Guessing of unknown atom radii	Y	Y	Y	Y				Y
Supports MOL2 format	Y							
Supports per atom CSA calculation	Y							
Supports BSA interface calculation	Y		Y	Y	Y			
Supports chain selections	Y	Y						
Supports per atom detailed output	Y	Y	Y	Y	Y	Y		Y
Source code available	Y	Y	Y		Y		Y	
Offline usage	Y	Y	Y		Y		Y	Y
Web server available	Y			Y		Y	Y	

Notes: Y, yes, supported by default; U, requires custom user settings; CSA, contact surface area; BSA, buried surface area.

References: FreeSASA (Mitternacht, 2016); NACCESS (Hubbard and Thornton, 1993); PISA (Krissinel and Henrick, 2007); CCP4 (Winn *et al.*, 2011); GetArea (Fraczkiewicz and Braun, 1998); DSSP (Touw *et al.*, 2015); MSMS (Sanner *et al.*, 1996).

1.9 Protein-DNA validation dataset

A non-redundant set of 245 protein-DNA complexes was obtained from our Protein-DNA Interface Database (PDIdb; Norambuena and Melo, 2010) and prepared as published before (Ribeiro *et al.*, 2015). Briefly, the amino acid sequences of the protein chains of 922 protein-DNA interface complexes were clustered with the computer program BLASTClust (Altschul *et al.*, 1990), according to a length coverage threshold of 90% and sequence identity of 70%. The resulting set is non-redundant in terms of the protein sequences and is available from our web site at http://melolab.org/pdidb/. A single structure (PDB code: 1qpi) was excluded from the analysis due to errors in the PDB file. All PDB files were processed with a script to remove atoms/residues/chains with alternative locations (altloc), keeping the location of higher occupancy, or in case of same occupancy, keeping the first position in sequential order.

1.10 Protein-ligand validation dataset

A protein-ligand dataset derived from PDBbind was prepared (Liu *et al.*, 2017). We used 290 protein-ligand complex structures defined by the authors as the ‘core set’, last updated November 2016. The core set is a non-redundant collection of protein-ligand complexes for which experimentally measured binding affinity data (K_d, K_i, and IC_{50}) are available. Ligands include small organic compounds and short peptides. Solvent atoms and atoms with alternate locations were removed, and the files were saved in the PDB format, and in addition in the Mol2 format with help of PyMOL (The PyMOL Molecular Graphics System, 2018).

1.11 Runtime comparison

dr_sasa is suitable for batch processing. SASA calculations for 290 protein-ligand complexes took 2.1 minutes on a 16-thread x86 notebook computer (AMD Ryzen 7 1700 @ 3.2 GHz), equivalent to 0.4 seconds per structure (3437.7 ± 2587.5 atoms per structure; Table S7). In addition, we have employed *dr_sasa* to calculate SASA and the interface contact surface area for 10,000 snapshots (PDB structures) extracted from a molecular dynamics trajectory of an "MarA-*micF*" protein-DNA complex (similar to PDB 1bl0; 1830 atoms). SASA calculations for the 10,000 snapshots took 36 min. on the 16-thread x86 notebook computer, equivalent to 0.2 s per structure. CSA calculations on the same dataset took 214 min. (1.3 s per structure).

Table S7. Runtime comparison of different SASA software tools.[1]

Sofware tool	*dr_sasa*	MSMS	NACCESS[2]	FreeSASA
Runtime	126.9 s	7.2 s	151.8 s	5.8 s

[1] Runtime comparison on a 16-thread x86 notebook computer (AMD Ryzen 7 1700 @ 3.2 GHz) with 290 protein-ligand complexes, SASA only

[2] Fails when running many instances in parallel, single core run

2 Benchmarks

2.1 Improved accuracy of contact surface area calculations by our modified Shrake-Rupley algorithm

Contact surface areas may be estimated approximately by calculating differences in SASA of artificially rearranged molecular objects. This approximate solution was implemented in the web server DNAproDB (Sagendorf *et al.*, 2017) and our own software tool PDIviz for the analysis of protein-DNA complexes (Ribeiro *et al.*, 2015). Here, we show that these approximations are rather rough and produced an average relative difference of -40% compared to *dr_sasa*'s direct calculation of interatomic contact surface areas (CSA).

To approximate CSA of protein atoms in contact with different DNA regions (e.g. protein surface buried by DNA bases), the following approach was implemented, which is based entirely on SASA calculations. First, an artificial protein-DNA complex is created by removing the atoms corresponding to certain DNA regions (bases, BB, major/minor groove) and its SASA is calculated. Next, SASA of the unmodified complex is calculated and subtracted from the SASA of the modified complex to give an estimate of CSA. Specifically:

CSA of protein atoms interacting with DNA bases:

CSA(protein←bases) = *SASA*(complex – DNA bases) – *SASA*(complex)

CSA of protein atoms interacting with DNA backbone:

CSA(protein←DNA backbone) = *SASA*(complex – DNA backbone) – *SASA*(complex)

CSA of protein atoms interacting with the major groove of DNA:

CSA(protein←major groove) = *SASA*(complex – major groove) – *SASA*(complex)

CSA of protein atoms interacting with the minor groove:

CSA(protein← minor groove) = *SASA*(complex – minor groove) – *SASA*(complex).

However, this is an approximate method and direct CSA calculation by our modified Shrake-Rupley algorithm should be more accurate. We determined CSA for protein atoms in contact with four different regions of the DNA double helix with *dr_sasa* (CSA mode 1) and compared these calculations with the results of the above approximate method estimated with (i) DNAproDB (Sagendorf *et al.*, 2017), which employs the software tool FreeSASA internally (Mitternacht, 2016), and with (ii) *dr_sasa* (SASA mode 0). We report the absolute (Figure S5, Figure S6, Table S8, and Table S9) and relative differences (Figure S7, Figure S8, Table S10, and Table S11) per structure.

We interpreted the results of this section as increased accuracy of *dr_sasa* due to its ability to calculate CSA directly from surface overlaps. Another possible explanation for the observed relative difference of -40% would be an error in *dr_sasa*'s CSA calculations. As a control experiment, we further demonstrate that all individual CSA's sum up to the total buried surface area (BSA), as expected (section 2.2). As BSA is typically estimated as differential SASA, we also show, as further control experiments, that SASA calculated by *dr_sasa* is similar to the values calculated by several other software tools (sections 2.3 and 2.4).

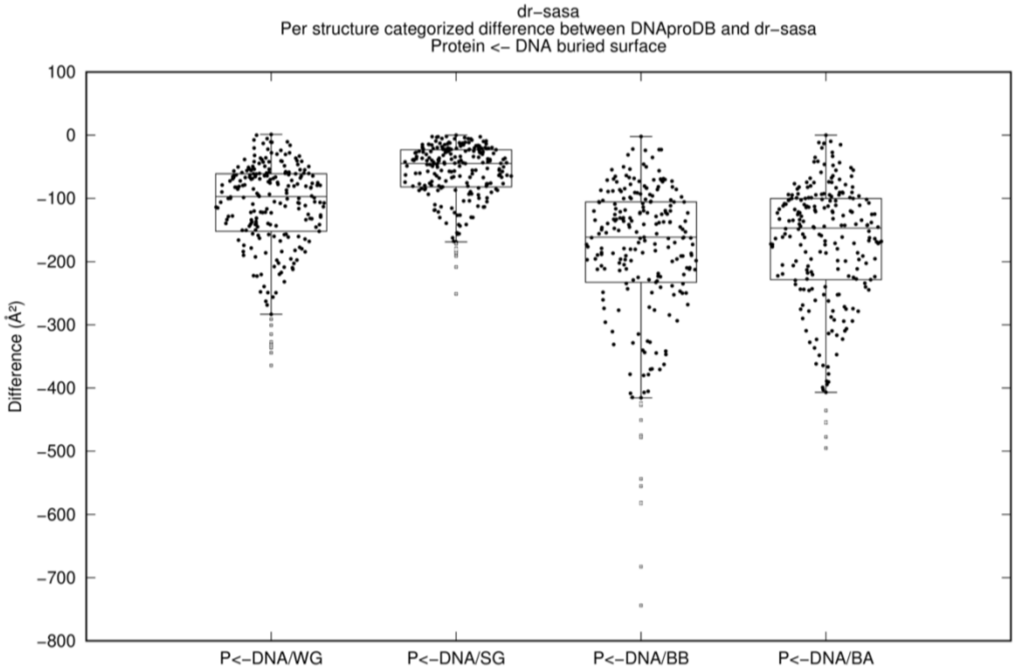

Figure S5. Comparison of DNAproDB vs. *dr_sasa* for calculating CSA, per structure. The protein-DNA dataset was employed (N = 226). 19 structures were removed due to incompatibilities with DNAproDB. Values were calculated as $Diff(CSA_i) = CSA_i^{DNAproDB} - CSA_i^{dr\_sasa}$, where $Diff_i(CSA)$ denotes the difference (possibly signed) of a CSA estimate of structure *i*. Box boundaries represent the 1st and 3rd quartile and box centers indicate the median. Whiskers are drawn at 1.5 IQR (interquartile range) and data points beyond these limits, if present, are indicated as crossed dots (outliers). Individual data points are plotted as swarms of filled dots. P: protein, WG: DNA major groove, SG: DNA minor groove, BB: DNA backbone, BA: DNA bases.

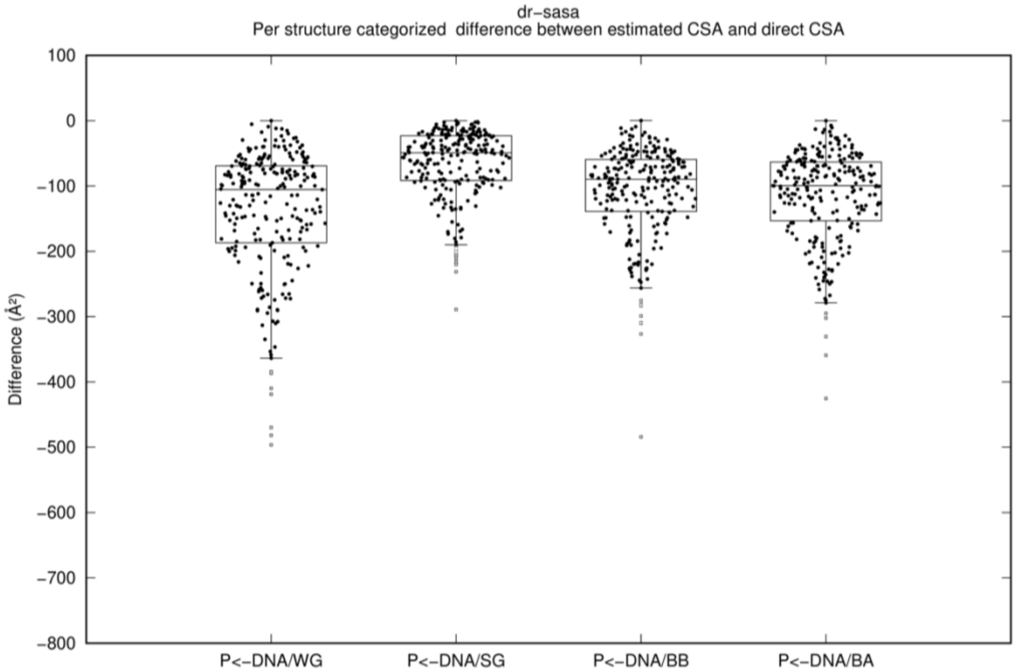

Figure S6. Comparison of *dr_sasa* (SASA mode) vs. *dr_sasa* (CSA mode) for calculating CSA, per structure. The protein-DNA dataset was employed (N = 245). Values were calculated as $Diff(CSA_i) = CSA_i^{dr\_sasa\ (SASA\ mode)} - CSA_i^{dr\_sasa\ (CSA\ mode)}$, where $Diff(CSA_i)$ denotes the difference (possibly signed) of a CSA estimate of structure *i*. Box plots were drawn as in Figure S5. WG: DNA major groove, SG: DNA minor groove, BB: DNA backbone, BA: DNA bases.

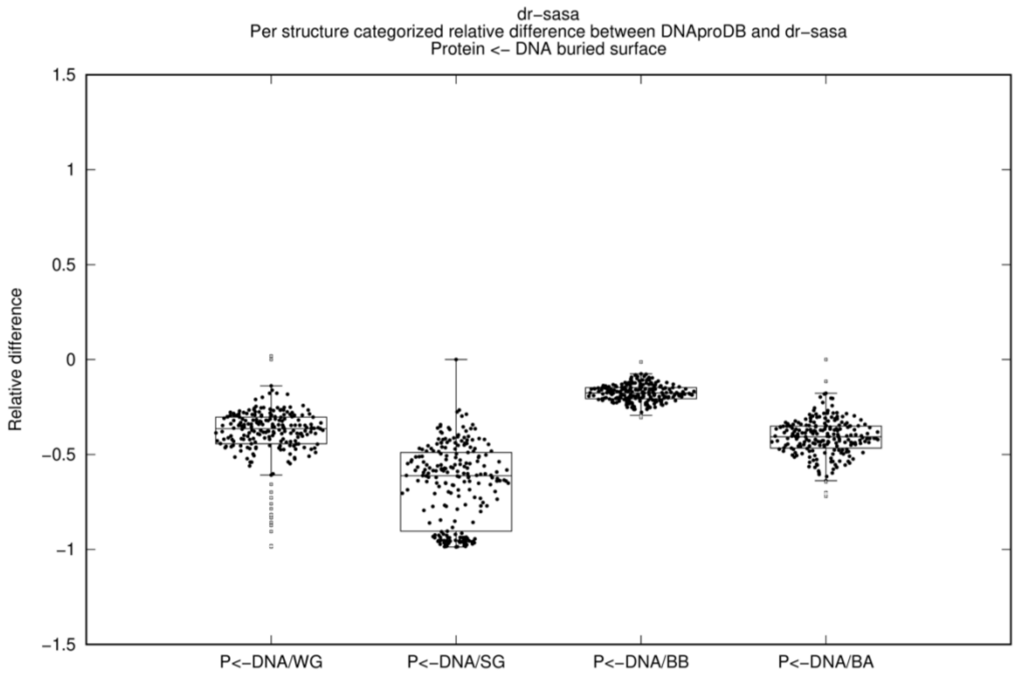

Figure S7. Comparison of DNAproDB vs. *dr_sasa* for calculating CSA, relative difference per structure. The protein-DNA dataset was employed (N = 226). 19 structures were removed due to incompatibilities with DNAproDB. Values were calculated as $Diff_{rel}(CSA_i) = \left((CSA_i^{DNAproDB} + 1) - (CSA_i^{dr\_sasa} + 1)\right)/(CSA_i^{dr\_sasa} + 1)$, where $Diff_{rel}(CSA_i)$ denotes the difference (possibly signed) of a CSA estimate of structure i. Box plots were drawn as in Figure S5. WG: DNA major groove, SG: DNA minor groove, BB: DNA backbone, BA: DNA bases.

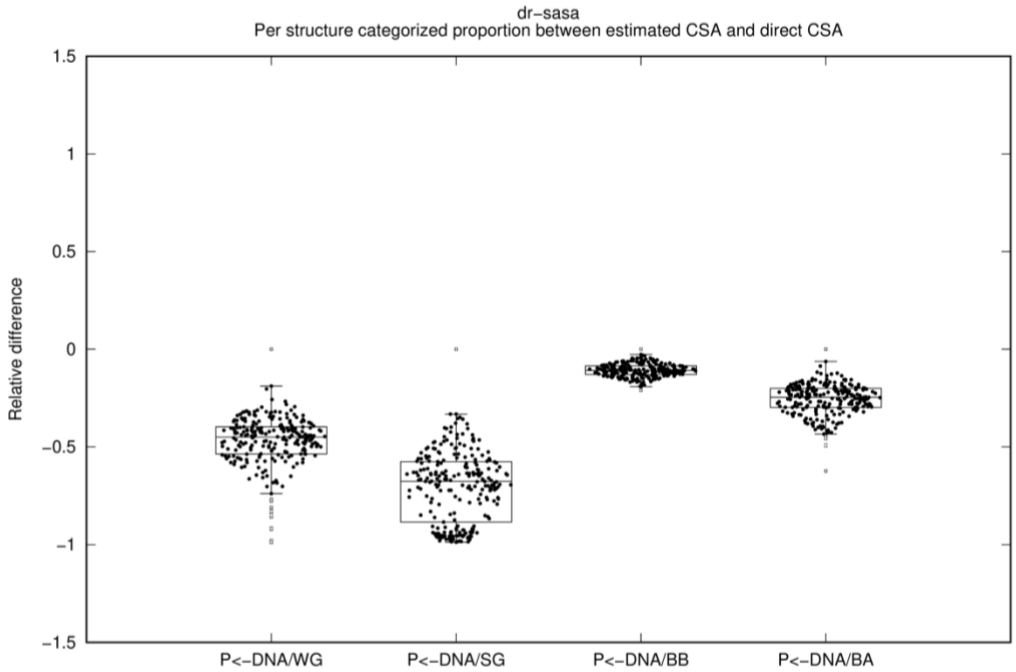

Figure S8. Comparison of *dr_sasa* (SASA mode) vs. *dr_sasa* (CSA mode) for calculating CSA, relative difference per structure. The protein-DNA dataset was employed (N = 245). Values were calculated as $Diff_{rel}(CSA_i) = \left((CSA_i^{dr\_sasa\ (SASA\ mode)} + 1) - (CSA_i^{dr\_sasa\ (CSA\ mode)} + 1)\right)/(CSA_i^{dr\_sasa\ (CSA\ mode)} + 1)$, where $Diff_{rel}(CSA_i)$ denotes the difference (possibly signed) of a CSA estimate of structure i. Box plots were drawn as in Figure S5. WG: DNA major groove, SG: DNA minor groove, BB: DNA backbone, BA: DNA bases.

Table S8. Comparison of DNAproDB vs. *dr_sasa* for calculating CSA, per structure.

Label	N	Quartile 1	Median	Quartile 3	Mean	Std. dev.
Protein←DNA-Major groove	226	-152.1	-97.2	-61.0	-114.3	74.3
Protein←DNA-Minor groove	226	-81.8	-44.8	-23.0	-59.5	47.0
Protein←DNA-Backbone	226	-232.8	-161.5	-105.5	-192.5	133.6
Protein←DNA-Bases	226	-228.7	-147.0	-100.1	-173.8	101.7

Values were calculated as $Diff(CSA_i) = CSA_i^{DNAproDB} - CSA_i^{dr\_sasa}$, where *Diff*(*CSA*$_i$) denotes the difference (possibly signed) of a BSA estimate of structure *i*. N is the total number of differences and std. dev. refers to the standard deviation.

Table S9. Comparison of *dr_sasa* (SASA mode) vs. *dr_sasa* (CSA mode) for calculating CSA, per structure.

Label	N	Quartile 1	Median	Quartile 3	Mean	Std. dev.
Protein←DNA-Major groove	245	-187.0	-105.4	-69.1	-137.8	95.9
Protein←DNA-Minor groove	245	-91.5	-49.1	-23.1	-67.0	55.1
Protein←DNA-Backbone	245	-139.1	-89.8	-59.3	-108.7	69.7
Protein←DNA-Bases	245	-153.2	-99.5	-63.4	-115.2	70.8

Values were calculated as $Diff(CSA_i) = CSA_i^{dr\_sasa\ (SASA\ mode)} - CSA_i^{dr\_sasa\ (CSA\ mode)}$, where *Diff*$_i$(*CSA*) denotes the difference (possibly signed) of a BSA estimate of structure *i*. N is the total number of differences and std. dev. refers to the standard deviation.

Table S10. Comparison of DNAproDB vs. *dr_sasa* for calculating CSA, relative differences per structure.

Label	N	Quartile 1	Median	Quartile 3	Mean	Std. dev.
Protein←DNA-Backbone	226	-44.3%	-36.4%	-30.3%	-39.3%	15.0%
Protein←DNA-Bases	226	-90.4%	-61.2%	-48.9%	-65.5%	20.9%
Protein←DNA-Major groove	226	-20.6%	-17.6%	-14.7%	-17.7%	4.6%
Protein←DNA-Minor groove	226	-46.6%	-40.7%	-35.0%	-41.2%	10.3%

Values were calculated as $Diff_{rel}(CSA_i) = \left(\left(CSA_i^{DNAproDB} + 1\right) - \left(CSA_i^{dr\_sasa} + 1\right)\right) / \left(CSA_i^{dr\_sasa} + 1\right)$, where *Diff*$_{rel}$(*CSA*$_i$) denotes the difference (possibly signed) of a CSA estimate of structure *i*. N is the total number of differences and std. dev. refers to the standard deviation.

Table S11. Comparison of *dr_sasa* (SASA mode) vs. *dr_sasa* (CSA mode) for calculating CSA, relative differences per structure.

Label	N	Quartile 1	Median	Quartile 3	Mean	Std. dev.
Protein←DNA-Backbone	245	-53.6%	-44.9%	-39.7%	-47.6%	14.0%
Protein←DNA-Bases	245	-88.4%	-67.6%	-57.5%	-70.1%	18.2%
Protein←DNA-Major groove	245	-12.9%	-10.9%	-8.5%	-10.7%	3.4%
Protein←DNA-Minor groove	245	-29.8%	-24.6%	-20.0%	-25.9%	8.4%

Values were calculated as $Diff_{rel}(CSA_i) = \left(\left(CSA_i^{dr\_sasa\ (SASA\ mode)} + 1\right) - \left(CSA_i^{dr\_sasa\ (CSA\ mode)} + 1\right)\right) / \left(CSA_i^{dr\_sasa\ (CSA\ mode)} + 1\right)$, where *Diff*$_{rel}$(*CSA*$_i$) denotes the difference (possibly signed) of a CSA estimate of structure *i*. N is the total number of differences and std. dev. refers to the standard deviation.

2.2 Validation of buried surface area calculations

In the previous section we show that dr_sasa's direct calculation of CSA is more accurate than estimating the contact surface from differential SASA. However, another possible explanation for the observed relative difference of -40% would be an error in *dr_sasa*'s CSA calculations. As a control experiment we demonstrate here that all individual CSA's sum up to the total buried surface area (BSA), as expected.

The buried surface area (BSA) of a molecule *i* forming a molecular complex is typically calculated by subtracting the SASA in the free state from the complexed state:

$$BSA = \Delta SASA = SASA_i^{free} - SASA_i^{complexed}.$$

However, *dr_sasa* calculates BSA as the sum of all individual contact surface areas (CSA) of all atoms participating in the interaction as:

$$BSA = \sum_{i=0}^{n} CSA_i$$

where *n* is the number of interacting atoms and CSA_i is the contact surface area of atom *i*. Our normalization scheme of contact surfaces shared between several atoms, as explained in section 1.2, ensures that this sum is equal to total BSA. This claim was validated on our protein-ligand dataset by calculating the difference between both methods per atom (Figure S9 and Table S12) and per structure (Figure S10 and Table S13). As expected, the average difference is zero with minor deviations due to limits in numerical floating point precision.

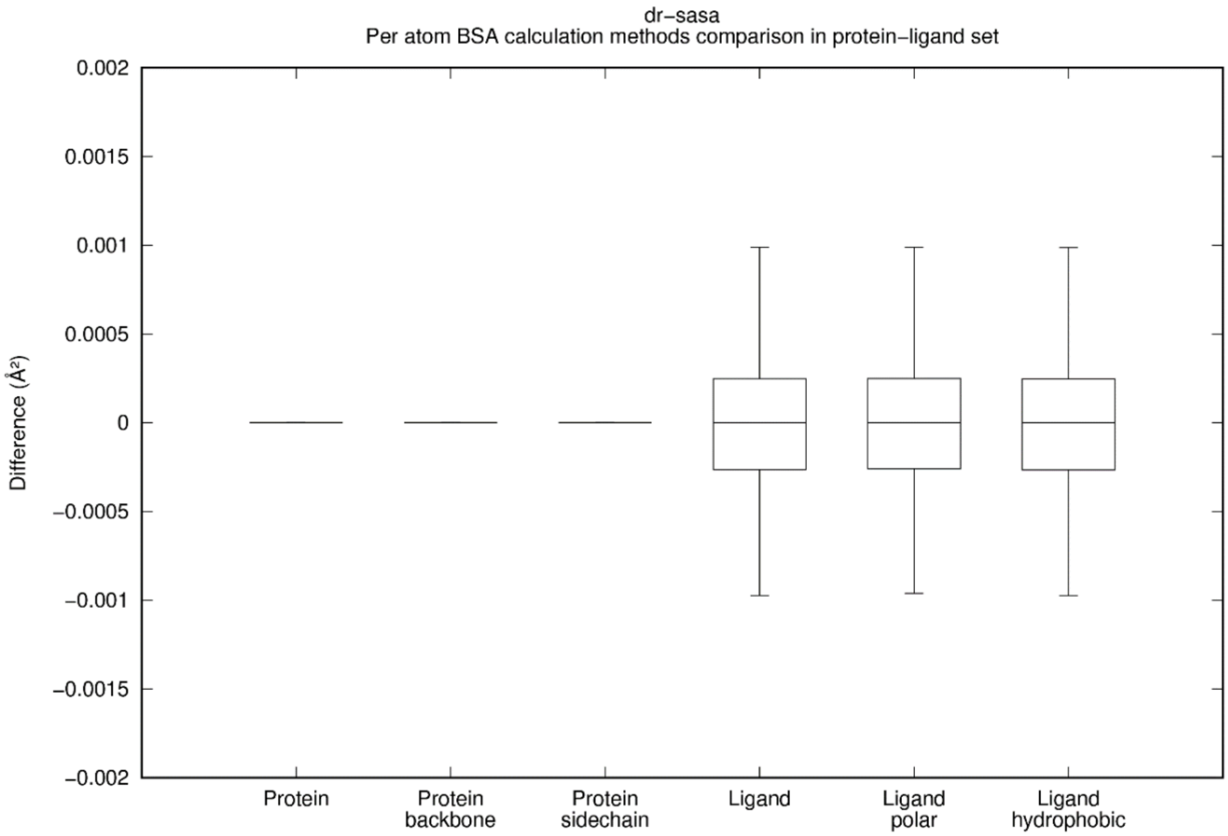

Figure S9. Validation of the BSA calculation, per atom. We compared the values obtained by calculating the total BSA as ΔSASA (subtracting the SASA of the molecular complex from the SASA of the free protein or ligand) with the sum of the contact surface areas (CSA) of *dr_sasa*. Differences were calculated as $Diff(BSA_i) = BSA_i^{\Delta SASA} - BSA_i^{\sum CSA}$, where *Diff* ($BSA_i$) denotes the difference (possibly signed) of a BSA estimate of atom *i*. Box boundaries represent the 1st and 3rd quartile and box centers indicate the median. Whiskers are drawn at 1.5 IQR (interquartile range) and data points beyond these limits, if present, are indicated as crossed dots (outliers). Individual data points (swarms) are omitted.

Table S12. Validation of the BSA calculation, per atom results.

	N	Quartile 1	Median	Quartile 3	Mean	Std. dev.
Protein	987957	0.000	0.000	0.000	0.000	0.000
Protein backbone	501266	0.000	0.000	0.000	0.000	0.000
Protein side chain	486691	0.000	0.000	0.000	0.000	0.000
Ligand	7070	0.000	0.000	0.000	0.000	0.000
Ligand polar	1841	0.000	0.000	0.000	0.000	0.000
Ligand hydrophobic	5229	0.000	0.000	0.000	0.000	0.000

Differences were calculated as $Diff(BSA_i) = BSA_i^{\Delta SASA} - BSA_i^{\sum CSA}$, where *Diff*($BSA_i$) denotes the difference (possibly signed) of a BSA estimate of atom *i*. N is the total number of differences and std. dev. refers to the standard deviation. All reported differences were zero with a precision of 3 decimal numbers.

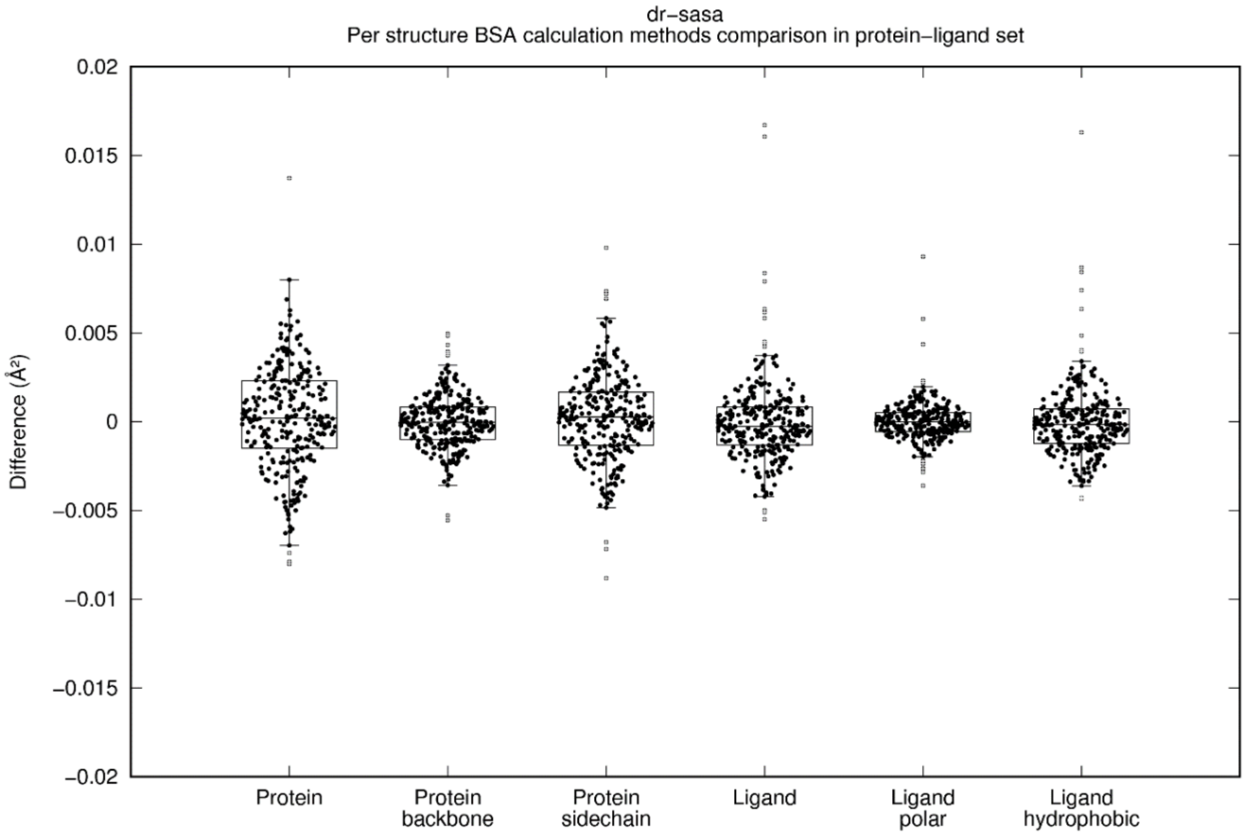

Figure S10. Validation of the BSA calculation, per structure. We compared the values obtained by calculating the total BSA as ΔSASA with the sum of the contact surface areas of *dr_sasa*. Differences were calculated as $Diff(BSA_i) = BSA_i^{\Delta SASA} - BSA_i^{\sum CSA}$, where *Diff* ($BSA_i$) denotes the difference (possibly signed) of a BSA estimate of structure *i*. The protein-ligand dataset was employed. Box plots were drawn as in Figure S5.

Table S13. Validation of the BSA calculation, per structure results.

	N	Quartile 1	Median	Quartile 3	Mean	Std. dev.
Protein	290	-0.001	0.000	0.002	0.000	0.003
Protein backbone	290	-0.001	0.000	0.001	0.000	0.002
Protein side chain	290	-0.001	0.000	0.002	0.000	0.002
Ligand	290	-0.001	0.000	0.001	0.000	0.002
Ligand polar	290	-0.001	0.000	0.001	0.000	0.001
Ligand hydrophobic	290	-0.001	0.000	0.001	0.000	0.002

Differences were calculated as $Diff(BSA_i) = BSA_i^{\Delta SASA} - BSA_i^{\sum CSA}$ where *Diff* (BSA_i) denotes the difference (possibly signed) of a BSA estimate of structure *i*. N is the total number of differences and std. dev. refers to the standard deviation.

2.3 Validation of solvent accessible surface area calculations for protein-ligand complexes

In section 2.1 we have shown that direct calculation of CSA is more accurate than relying on rough differential SASA estimates, and in section 2.2 we have demonstrated that these results are not likely caused by an error in *dr_sasa*, as all individual atomic CSA's indeed summed up to the total BSA.

Here, we further show that *dr_sasa*'s calculation of plain SASA is similar to the values obtained by the software tools NACCESS (Hubbard and Thornton, 1993), MSMS (Sanner *et al.*, 1996), and FreeSASA (Mitternacht, 2016). SASA was calculated for 290 protein-ligand complexes provided in the PDB format with the three software tools and the difference in surface area to *dr_sasa* was analyzed per atom (Figure S11 and Table S14) and per structure (Figure S12 and Table S15). The average difference per structure compared to NACCESS was -0.699 ± 12.015 Å (mean ± standard deviation) and was 2.265 ± 12.584 Å for FreeSASA. However, for MSMS we obtained a large difference of -130.691 ± 316.274 Å^2 per structure. As an explanation, we observed that in some cases MSMS determined a zero value for SASA for atoms which were solvent exposed as by *dr_sasa*/NACCESS/FreeSASA. In Table S16 we compared total SASA of 13 protein-ligand complexes calculated by all four software tools. These 13 protein-ligand complexes had large MSMS vs. *dr_sasa* differences (< -500 Å^2). 95% of these can be explained by atoms for which MSMS calculated an exposed surface of 0 Å^2 but were detected solvent exposed by *dr_sasa*. We conclude that these large deviations are a peculiarity of MSMS, as results of *dr_sasa*, NACCESS and FreeSASA were similar. In addition, these large deviations were outliers related to very large structures, as we obtained a low relative difference of < 1% per structure (Figure S13 and Table S17).

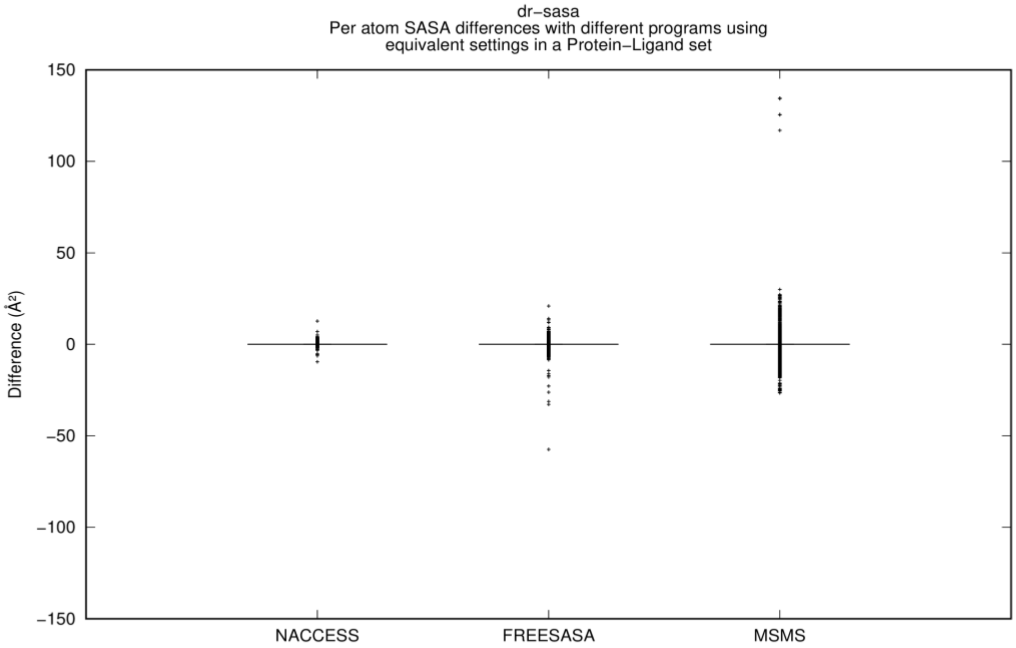

Figure S11. Comparison of SASA values of *dr_sasa* and three other SASA tools, per atom. The protein-ligand dataset was employed. We observed that MSMS determined a zero value for SASA for some atoms which were solvent-exposed, hence the large number of outliers. Differences were calculated as $Diff(SASA_i) = SASA_i^{NACCESS/FreeSASA/MSMS} - SASA_i^{dr\_sasa}$, where *Diff*($SASA_i$) denotes the (possibly signed) difference of a SASA estimate of atom *i*. Box plots shown here were drawn as in Figure S5.

Table S14. Comparison of SASA values of *dr_sasa* and three other SASA tools for the protein-ligand dataset, per atom (Å^2).

	N	Quartile 1	Median	Quartile 3	Mean	Std. dev.
NACCESS	995027	-0.010	0.000	0.012	0.000	0.166
FreeSASA	995027	-0.010	0.000	0.010	0.001	0.210
MSMS	995027	-0.006	0.000	0.001	-0.038	0.681

Differences were calculated as $Diff(SASA_i) = SASA_i^{NACCESS/FreeSASA/MSMS} - SASA_i^{dr\_sasa}$, where *Diff*($SASA_i$) denotes the (possibly signed) difference of a SASA estimate of atom *i*. N is the total number of differences and std. dev. refers to the standard deviation.

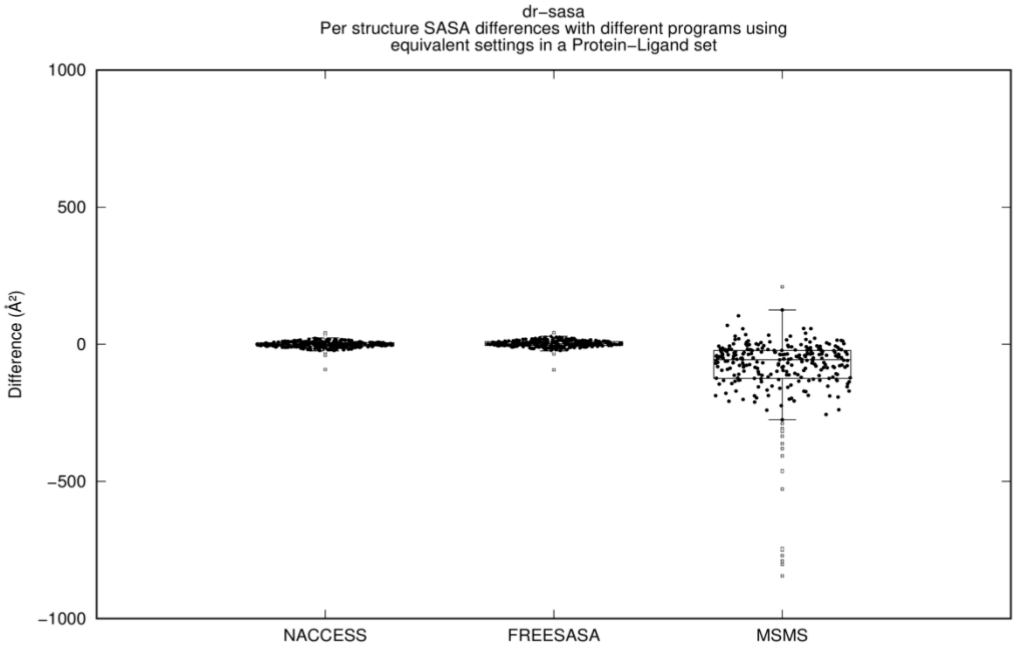

Figure S12. Comparison of SASA values of *dr_sasa* and three other SASA tools, per structure. The protein-ligand dataset was employed. For a discussion of the outliers, please refer to the main text. Differences were calculated as $Diff(SASA_i) = SASA_i^{NACCESS/FreeSASA/MSMS} - SASA_i^{dr\_sasa}$, where $Diff(SASA_i)$ denotes the (possibly signed) difference of a SASA estimate of structure *i*. Box plots shown here were drawn as in Figure S5.

Table S15. Comparison of SASA values of *dr_sasa* and three other SASA tools for the protein-ligand dataset, per structure (Å^2).

	N	Quartile 1	Median	Quartile 3	Mean	Std. dev.
NACCESS	290	-6.323	-0.842	5.890	-0.699	12.015
FreeSASA	290	-4.250	1.540	10.080	2.265	12.584
MSMS	290	-124.175	-56.229	-22.450	-130.691	316.274

Differences were calculated as $Diff(SASA_i) = SASA_i^{NACCESS/FreeSASA/MSMS} - SASA_i^{dr\_sasa}$, where $Diff(SASA_i)$ denotes the (possibly signed) difference of a SASA estimate of structure *i*. N is the total number of differences and std. dev. refers to the standard deviation.

Table S16. Comparison of MSMS calculation outliers with < -500 Å^2 difference with *dr_sasa*.

	Total area				All atoms*	Atoms with zero SASA*
PDB ID	*dr_sasa*	NACCESS	FreeSASA	MSMS	diff(MSMS–*dr_sasa*)	diff(MSMS–*dr_sasa*)
3ebp	57650.5	57627.8	57643.4	55143.7	-2506.8	-2406.0
4ciw	56974.6	56882.8	56881.5	54491.1	-2483.6	-2363.6
3l7b	57942.8	57938.5	57937.3	55572.8	-2370.0	-2365.0
3syr	58510.6	58535.5	58536.1	56309.2	-2201.4	-2090.3
4eky	58175.1	58178.7	58177.0	56505.2	-1669.9	-1555.9
1ps3	36031.4	36027.5	36028.3	35186.9	-844.5	-803.0
3g2n	31135.0	31118.8	31118.3	30332.6	-802.4	-756.1
3ejr	36228.7	36238.0	36238.7	35438.9	-789.8	-775.1
2ymd	80796.2	80809.8	80809.7	80025.8	-770.3	-692.3
3dx1	35765.1	35769.4	35770.4	35015.2	-749.9	-738.1
3d4z	35497.3	35515.1	35514.0	34751.2	-746.1	-707.0
3dx2	35753.3	35782.0	35782.3	35009.1	-744.1	-721.6
3f3c	36899.1	36870.7	36870.5	36371.1	-527.9	-444.6

* Difference calculated as SASA(MSMS) – SASA(*dr_sasa*) for all atoms and for atoms for which MSMS calculated an exposed surface of 0 Å^2.

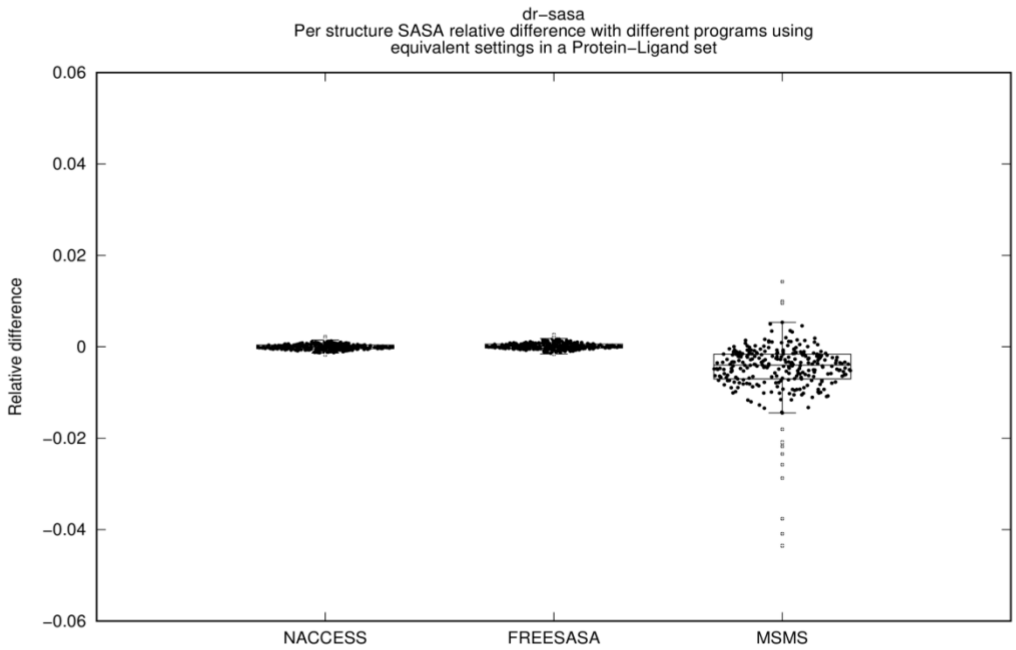

Figure S13. Comparison of SASA values of *dr_sasa* and three other SASA tools, relative difference per structure. The protein-ligand dataset was employed. Values were calculated as $Diff_{rel}(SASA_i) = \left((SASA_i^{NACCESS/FreeSASA/MSMS} + 1) - (SASA_i^{dr\_sasa} + 1)\right) / (SASA_i^{dr\_sasa} + 1)$, where $Diff_{rel}(SASA_i)$ denotes the relative difference of a SASA estimate of structure *i*. Box plots were drawn as in Figure S5.

Table S17. Comparison of SASA values of *dr_sasa* and three other SASA tools for the protein-ligand dataset, relative difference per structure.

	N	Quartile 1	Median	Quartile 3	Mean	Std. dev.
NACCESS	290	-0.04%	-0.01%	0.04%	0.00%	0.06%
FreeSASA	290	-0.03%	0.01%	0.06%	0.02%	0.07%
MSMS	290	-0.70%	-0.40%	-0.16%	-0.51%	0.66%

Values were calculated as $Diff_{rel}(SASA_i) = \left((SASA_i^{NACCESS/FreeSASA/MSMS} + 1) - (SASA_i^{dr\_sasa} + 1)\right) / (SASA_i^{dr\_sasa} + 1)$, where $Diff_{rel}(SASA_i)$ denotes the relative difference of a SASA estimate of structure *i*. N is the total number of differences and std. dev. refers to the standard deviation.

2.4 Validation of solvent accessible surface area calculations for protein-DNA complexes

We further validated plain SASA calculations for 245 protein-DNA complexes with *dr_sasa* and NACCESS. We report the difference in surface area per atom (Figure S14 and Table S18) and per structure (Figure S15 and Table S19). We obtained a low overall difference of -0.074 ± 1.804 Å^2 (mean ± standard deviation) per structure.

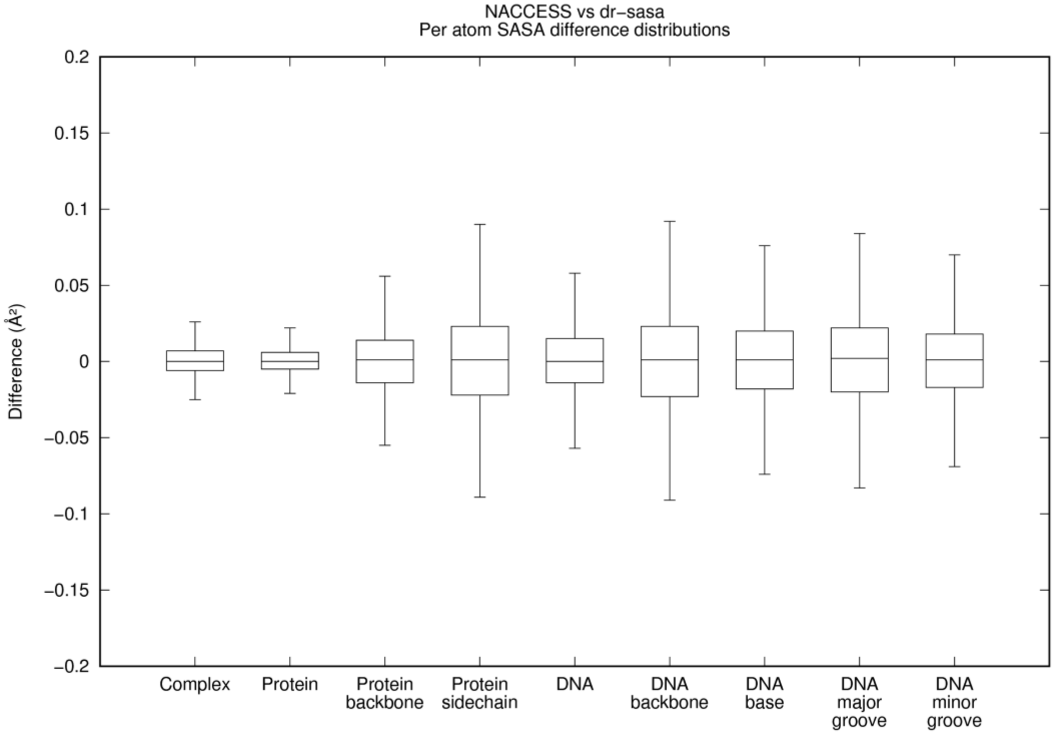

Figure S14. Difference of estimated SASA per atom between NACCESS and *dr_sasa*. SASA for all atoms of the protein-DNA set was calculated with NACCESS 2.1.1 and *dr_sasa*. Differences were calculated as $Diff(SASA_i) = SASA_i^{NACCESS} - SASA_i^{dr\_sasa}$, where *Diff*($SASA_i$) denotes the (possibly signed) difference of a SASA estimate of atom *i*. Box plots were drawn as in Figure S9.

Table S18. Differences of SASA estimations per atom (Å^2) between NACCESS and *dr_sasa*.

	N	Quartile 1	Median	Quartile 3	Mean	Std. dev.
Complex	750911	-0.006	0.000	0.007	0.001	0.381
Protein	563670	-0.005	0.000	0.006	0.000	0.104
Protein backbone	136377	-0.014	0.001	0.014	0.000	0.049
Protein sidechain	205788	-0.022	0.001	0.023	-0.001	0.167
DNA	187241	-0.014	0.000	0.015	0.000	0.036
DNA backbone	95564	-0.023	0.001	0.023	0.000	0.043
DNA base	49935	-0.018	0.001	0.020	0.000	0.038
DNA major groove	31165	-0.020	0.002	0.022	0.000	0.040
DNA minor groove	17098	-0.017	0.001	0.018	0.000	0.034

Differences were calculated as $Diff(SASA_i) = SASA_i^{NACCESS} - SASA_i^{dr\_sasa}$, where *Diff*($SASA_i$) denotes the (possibly signed) difference of a SASA estimate of atom *i*. N is the total number of differences and std. dev. refers to the standard deviation.

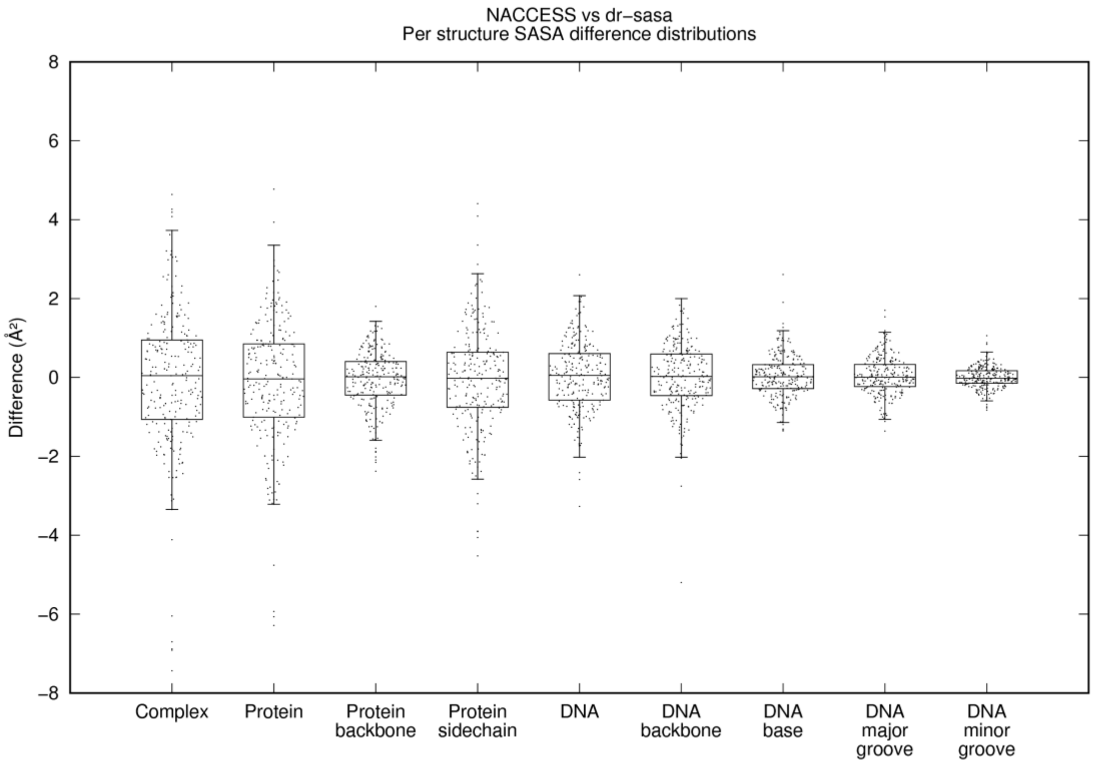

Figure S15. Difference of estimated SASA per structure between NACCESS and *dr_sasa*. SASA for all structures (protein-DNA complexes) in the non-redundant set was calculated with NACCESS 2.1.1 and *dr_sasa*. Differences were calculated as $Diff(SASA_i) = SASA_i^{NACCESS} - SASA_i^{dr\_sasa}$, where Diff(SASA) denotes the (possibly signed) difference of a SASA estimate of structure i. Box plots were drawn as in Figure S5.

Table S19. Differences of SASA estimations per structure (Å^2) between NACCESS and *dr_sasa*.

	N	Quartile 1	Median	Quartile 3	Mean	Std. dev.
Complex	245	-1.065	0.047	0.947	-0.074	1.804
Protein	245	-1.008	-0.039	0.850	-0.108	1.582
Protein backbone	245	-0.446	0.019	0.406	-0.055	0.720
Protein sidechain	245	-0.756	-0.018	0.637	-0.053	1.322
DNA	245	-0.573	0.052	0.605	0.039	0.889
DNA backbone	245	-0.460	0.024	0.592	0.007	0.856
DNA base	245	-0.283	0.019	0.324	0.032	0.542
DNA major groove	245	-0.229	0.004	0.328	0.039	0.476
DNA minor groove	245	-0.148	-0.018	0.172	-0.004	0.273

Differences were calculated as $Diff(SASA_i) = SASA_i^{NACCESS} - SASA_i^{dr\_sasa}$, where Diff(SASA$_i$) denotes the (possibly signed) difference of a SASA estimate of structure i. N is the total number of differences and std. dev. refers to the standard deviation.

2.5 Effect of different vdW radii definitions on SASA calculation

Structures provided in PDB format use a different vdW radii definition than structures provided in Mol2 format (c.f. section 1.3). To estimate the effect of the different vdW radii definitions, we calculated SASA for our protein-ligand set provided as PDB files and Mol2 files (converted from PDB by PyMOL) and calculated the difference by atom (Figure S16 and Table S20) and structure (Figure S17 and Table S21), and the relative difference per structure (Figure S18 and Table S22). We observed a low relative difference of 1% per protein, and a relative difference of 10% per ligand. This is an expected result, since the atom typing and vdW radii used for the Mol2 format allows for a more fine-grained description of ligand atoms.

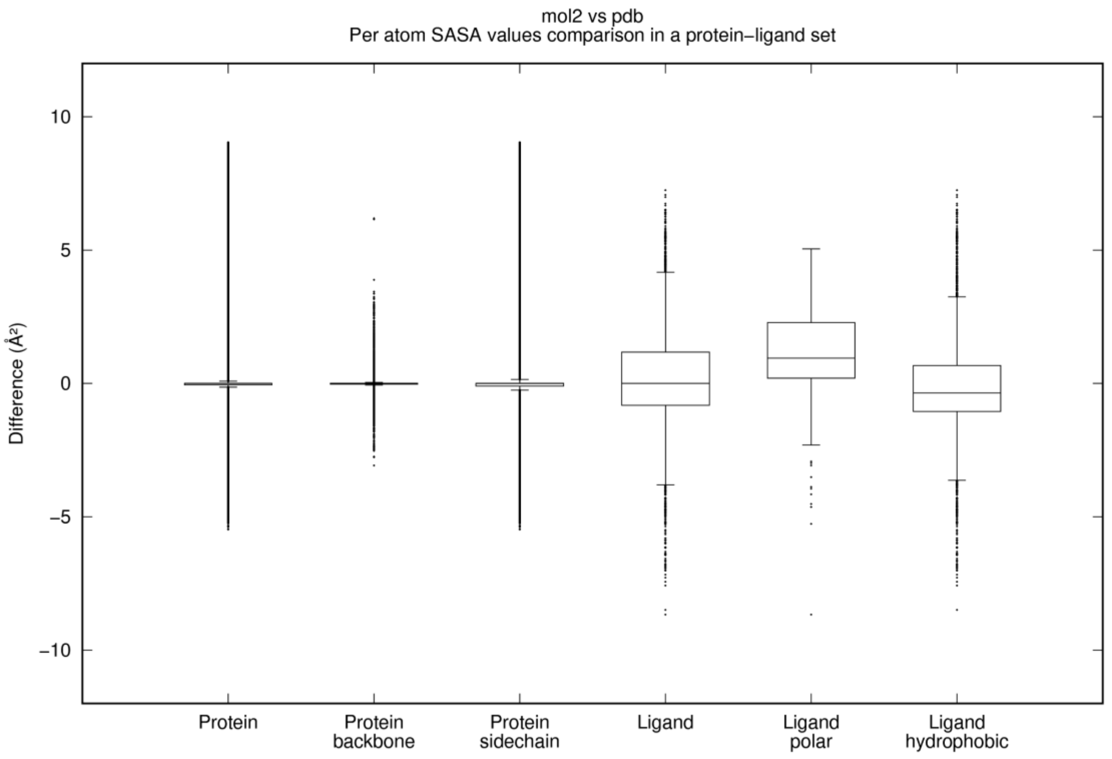

Figure S16. Per atom comparison of SASA for different vdW radii definitions. The structures of the protein-ligand set were provided either in Mol2 or in PDB format. Values were calculated as $Diff(SASA_i) = SASA_i^{Mol2} - SASA_i^{PDB}$, where *Diff*(*SASA*$_i$) denotes the difference (possibly signed) of a SASA estimate of atom *i*. Box plots were drawn as in Figure S5.

Table S20. Per atom comparison of SASA for different vdW radii definitions.

Label	N	Quartile 1	Median	Quartile 3	Mean	Std. dev.
Protein	987957	-0.056	0.000	0.000	0.001	0.640
Protein backbone	501266	-0.024	0.000	0.000	-0.017	0.137
Protein side chain	486691	-0.100	0.000	0.000	0.019	0.901
Ligand	7070	-0.821	0.000	1.176	0.203	1.850
Ligand polar	1841	0.199	0.951	2.281	1.261	1.475
Ligand hydrophobic	5229	-1.052	-0.357	0.669	-0.169	1.825

The structures of the protein-ligand set were provided either in Mol2 or in PDB format. Values were calculated as $Diff(SASA_i) = SASA_i^{Mol2} - SASA_i^{PDB}$, where *Diff*(*SASA*$_i$) denotes the difference (possibly signed) of a SASA estimate of atom *i*. N is the total number of differences and std. dev. refers to the standard deviation.

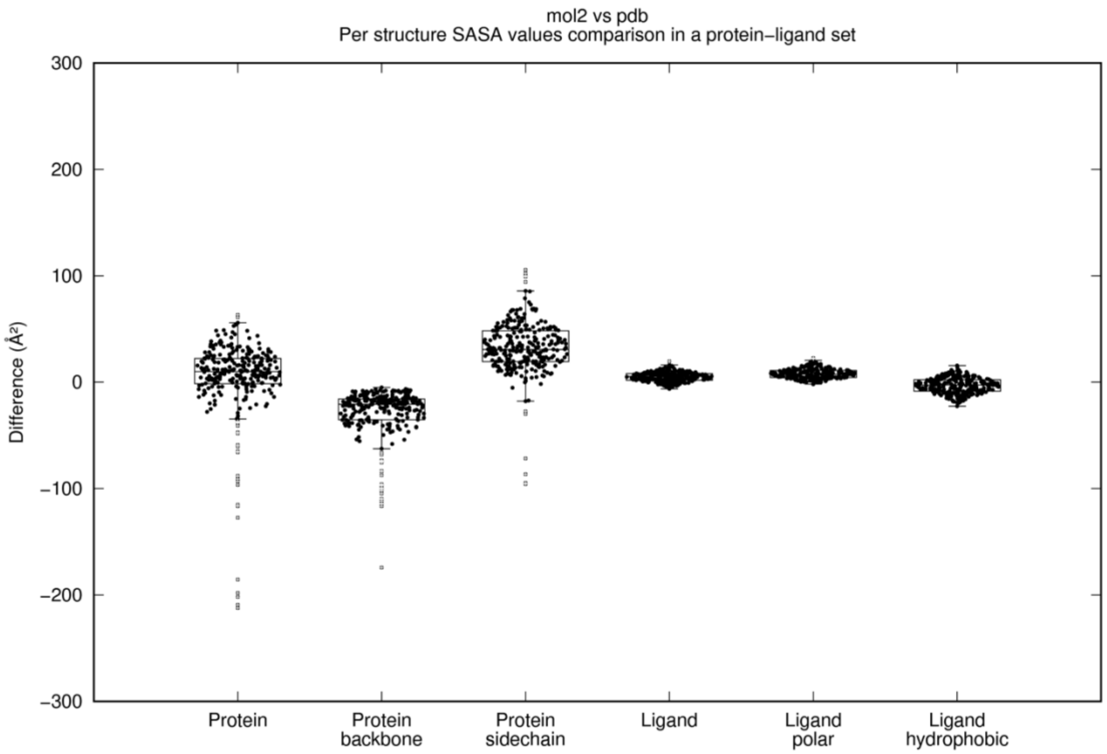

Figure S17. Per structure comparison of SASA for different vdW radii definitions. The structures of the protein-ligand set were provided either in Mol2 or in PDB format. Values were calculated as $Diff(SASA_i) = SASA_i^{Mol2} - SASA_i^{PDB}$, where *Diff*(*SASA*$_i$) denotes the difference (possibly signed) of a SASA estimate of structure *i*. Box plots were drawn as in Figure S5.

Table S21. Per structure comparison of SASA for different vdW radii definitions.

Label	N	Quartile 1	Median	Quartile 3	Mean	Std. dev.
Protein	290	-1.367	9.935	22.283	2.932	38.736
Protein backbone	290	-35.336	-20.788	-15.756	-29.211	24.161
Protein side chain	290	19.381	30.754	48.382	32.142	24.936
Ligand	290	1.837	5.361	8.129	4.950	4.474
Ligand polar	290	4.388	7.461	11.006	8.003	4.838
Ligand hydrophobic	290	-8.458	-2.742	2.298	-3.054	7.343

The structures of the protein-ligand set were provided either in Mol2 or in PDB format. Values were calculated as $Diff(SASA_i) = SASA_i^{Mol2} - SASA_i^{PDB}$, where *Diff*(*SASA*$_i$) denotes the difference (possibly signed) of a SASA estimate of structure *i*. N is the total number of differences and std. dev. refers to the standard deviation.

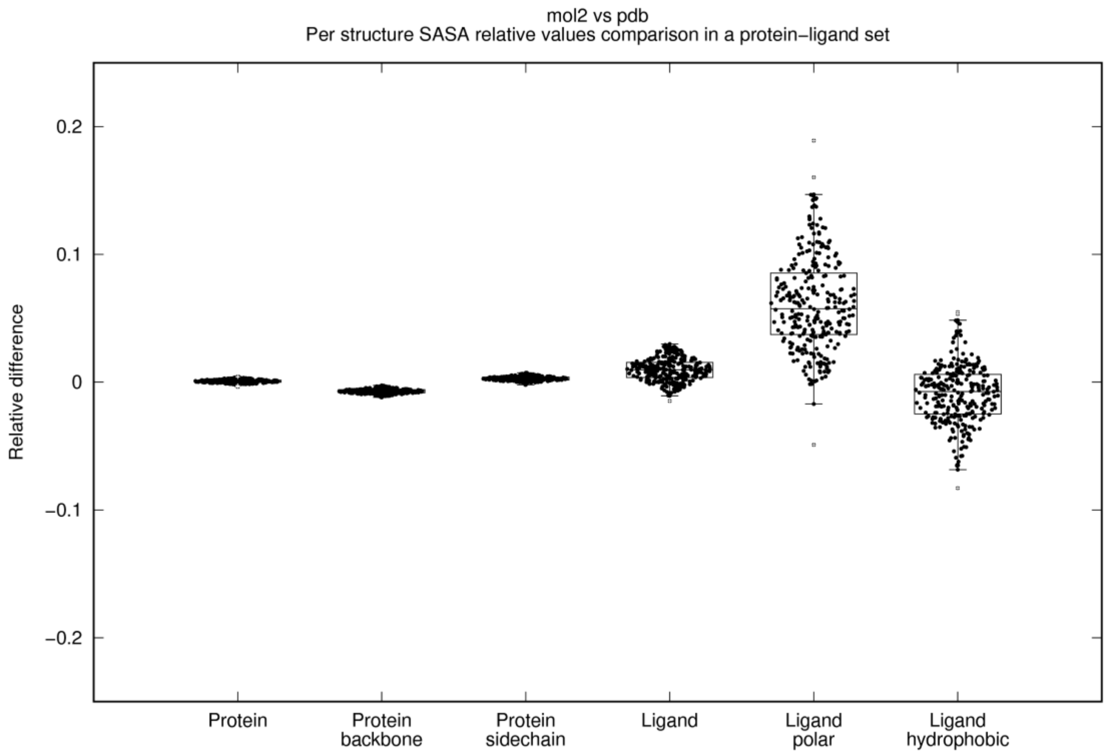

Figure S18. Relative difference per structure of SASA for different vdW radii definitions. The structures of the protein-ligand set were provided either in Mol2 or in PDB format. Values were calculated as $Diff_{rel}(SASA_i) = \left((SASA_i^{Mol2} + 1) - (SASA_i^{PDB} + 1)\right)/(SASA_i^{PDB} + 1)$, where $Diff_{rel}(SASA_i)$ denotes the (possibly signed) relative difference of a SASA estimate of structure *i*. Box plots were drawn as in Figure S5.

Table S22. Relative difference per structure of SASA for different vdW radii definitions.

Label	N	Quartile 1	Median	Quartile 3	Mean	Std. dev.
Protein	290	0.00%	0.10%	0.20%	0.10%	0.10%
Protein backbone	290	-0.80%	-0.70%	-0.60%	-0.70%	0.20%
Protein side chain	290	0.20%	0.30%	0.40%	0.30%	0.20%
Ligand	290	0.40%	1.00%	1.60%	1.00%	0.90%
Ligand polar	290	3.70%	5.80%	8.50%	6.10%	3.70%
Ligand hydrophobic	290	-2.50%	-0.70%	0.60%	-0.90%	2.30%

The structures of the protein-ligand set were provided either in Mol2 or in PDB format. Values were calculated as $Diff_{rel}(SASA_i) = \left((SASA_i^{Mol2} + 1) - (SASA_i^{PDB} + 1)\right)/(SASA_i^{PDB} + 1)$, where $Diff_{rel}(SASA_i)$ denotes the (possibly signed) relative difference of a SASA estimate of structure *i*. N is the total number of differences and std. dev. refers to the standard deviation.

2.6 Calculation of CSA without requiring that the contact surfaces are solvent accessible

Surface-based interaction analysis of protein-ligand complexes is complicated by the fact that many ligand binding sites are deep cavities that may be poorly accessible by the rolling ball approximation. In these cases, the actual protein-ligand contact surface area will be underestimated if the binding pockets are poorly solvent exposed. We therefore implemented a variation of our CSA calculation, in which atoms are not required to be solvent accessible (mode 4 of *dr_sasa*). We compared the area difference of this mode against solvent exposed CSA calculations (mode 1 of *dr_sasa*) on our protein-ligand dataset. Results are reported per atom (Figure S19 and Table S23) and per structure (Figure S20 and Table S24). Our results indicate that the relative difference in BSA without the requirement that atoms were solvent exposed was, on average, 10 times larger per protein structure compared to BSA of mode 1 (Figure S21 and Table S25).

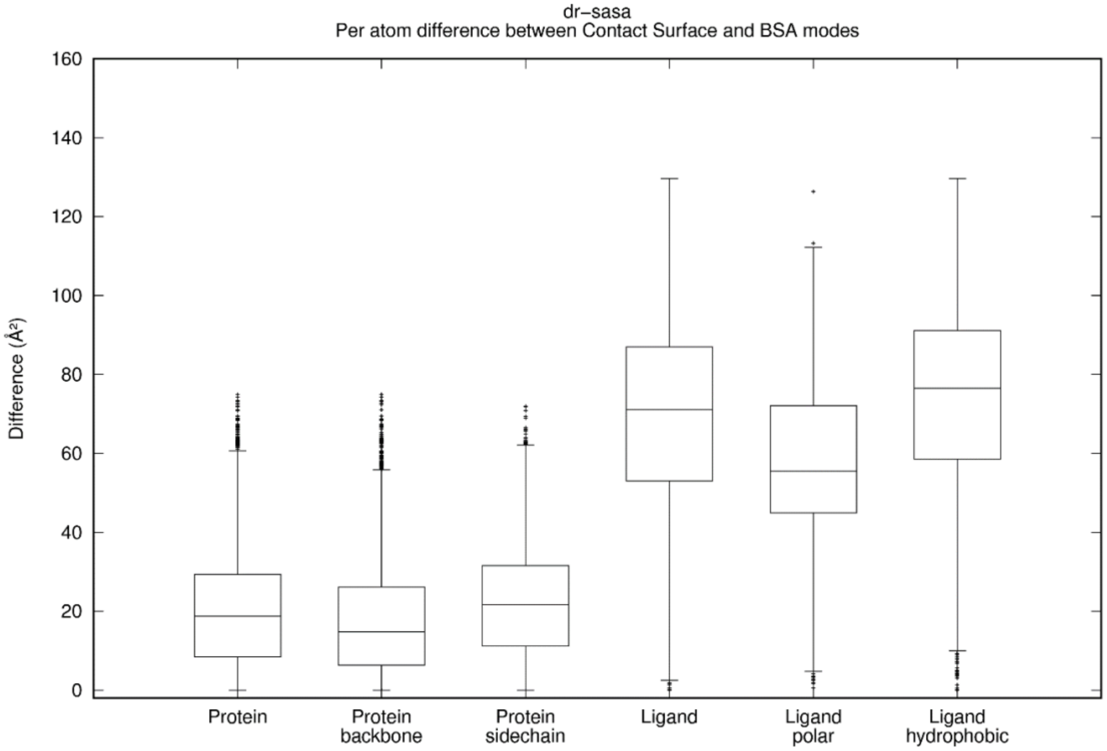

Figure S19. Comparison of BSA without and with the requirement that atoms are solvent exposed, per atom. The protein-ligand dataset was employed. Values were calculated as $Diff(BSA_i) = BSA_i^{dr\_sasa\ mode\ 4} - BSA_i^{dr\_sasa\ mode\ 1}$, where *Diff* ($BSA_i$) denotes the difference (possibly signed) of a BSA estimate of atom *i*. Box plots were drawn as in Figure S9.

Table S23. Comparison of BSA without and with the requirement that atoms are solvent exposed, per atom.

	N	Quartile 1	Median	Quartile 3	Mean	Std. dev.
Protein	35188	8.412	18.777	29.373	19.783	13.166
Protein backbone	15453	6.342	14.790	26.171	17.045	12.632
Protein side chain	19735	11.158	21.593	31.568	21.928	13.179
Ligand	7035	53.057	71.110	86.966	69.726	23.208
Ligand polar	1825	44.944	55.494	72.087	57.011	19.841
Ligand hydrophobic	5210	58.575	76.489	91.144	74.181	22.649

Values were calculated as $Diff(BSA) = BSA_i^{dr\_sasa\ mode\ 4} - BSA_i^{dr\_sasa\ mode\ 1}$, where *Diff*(*BSA*) denotes the (possibly signed) difference of a BSA estimate of atom *i*. N is the total number of differences and std. dev. refers to the standard deviation. The protein-ligand dataset was employed.

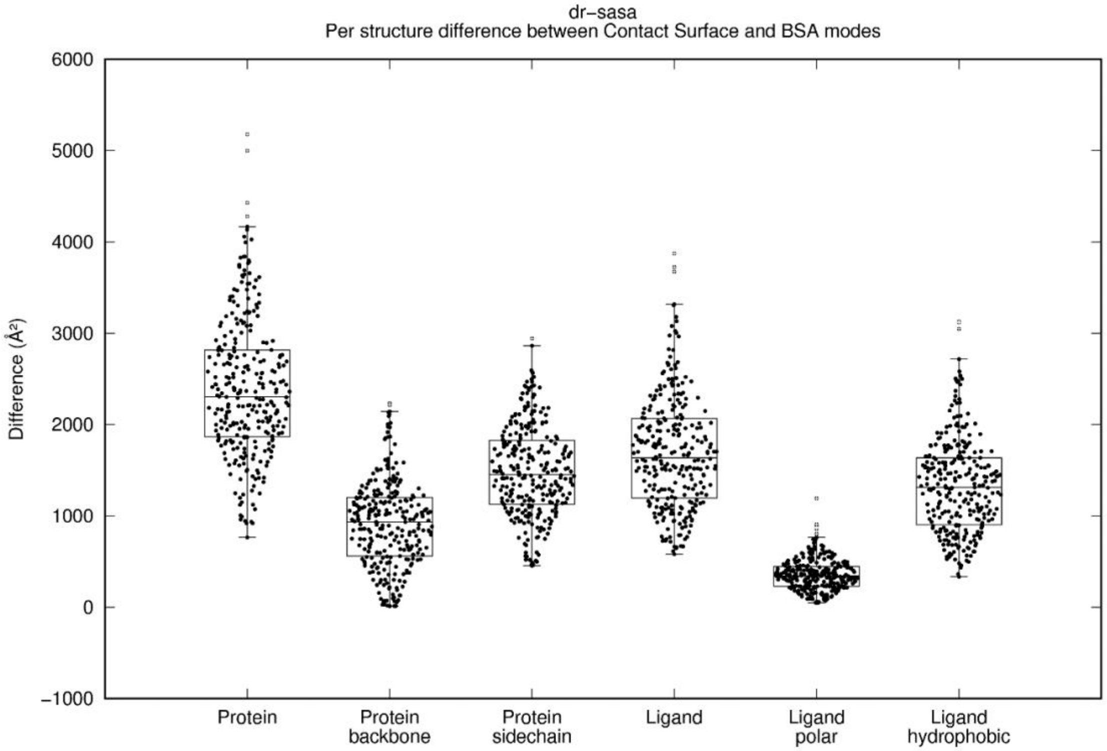

Figure S20. Comparison of BSA without and with the requirement that atoms are solvent exposed, per structure. The protein-ligand dataset was employed. Values were calculated as $Diff(BSA_i) = BSA_i^{dr\_sasa\ mode\ 4} - BSA_i^{dr\_sasa\ mode\ 1}$, where *Diff* ($BSA_i$) denotes the difference (possibly signed) of a BSA estimate of structure i. Box plots were drawn as in Figure S5.

Table S24. Comparison of BSA without and with the requirement that atoms are solvent exposed, per structure.

	N	Quartile 1	Median	Quartile 3	Mean	Std. dev.
Protein	290	1867.590	2305.550	2816.820	2400.460	762.016
Protein backbone	290	560.353	933.095	1203.360	908.251	478.055
Protein side chain	290	1127.060	1453.630	1828.150	1492.210	502.082
Ligand	290	1196.400	1635.160	2068.250	1691.470	617.718
Ligand polar	290	228.912	341.846	446.369	358.773	174.997
Ligand hydrophobic	290	901.551	1312.340	1636.820	1332.690	523.824

Values were calculated as $Diff(BSA_i) = BSA_i^{dr\_sasa\ mode\ 4} - BSA_i^{dr\_sasa\ mode\ 1}$, where *Diff*($BSA_i$) denotes the (possibly signed) difference of a BSA estimate of structure i. N is the total number of differences and std. dev. refers to the standard deviation. The protein-ligand dataset was employed.

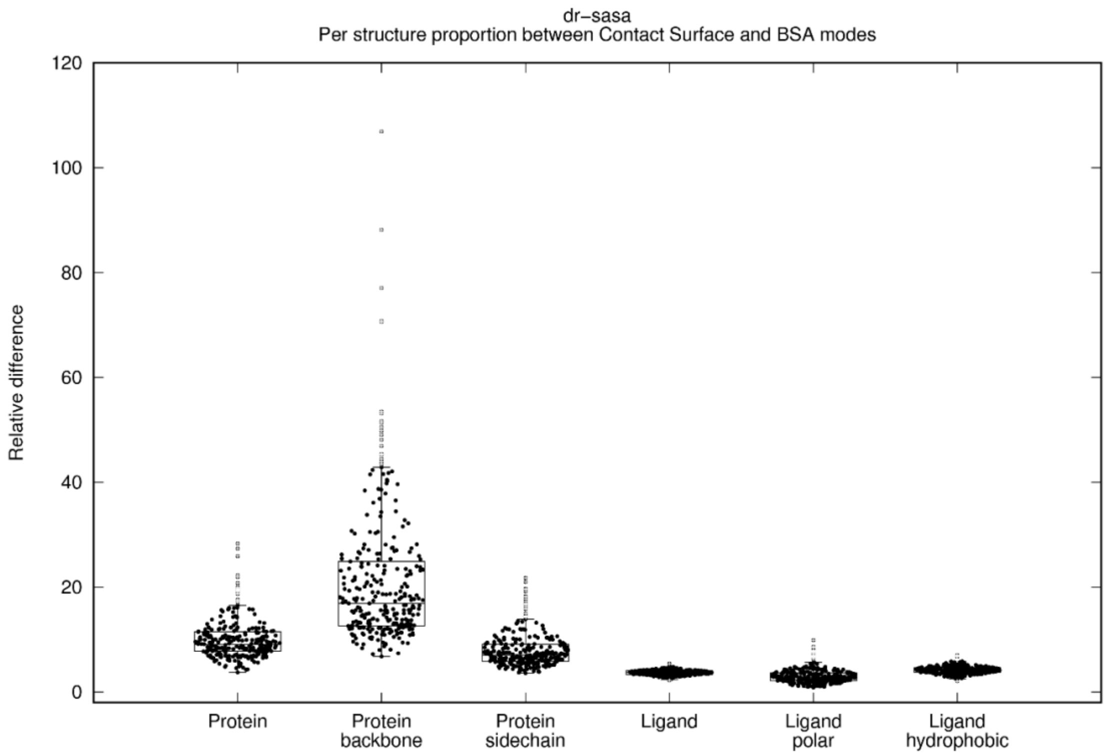

Figure S21. Comparison of BSA without and with the requirement that atoms are solvent exposed, relative difference per structure. The protein-ligand dataset was employed. Values were calculated as $Diff_{rel}(BSA_i) = \left((BSA_i^{Mode\ 4} + 1) - (BSA_i^{Mode\ 1} + 1)\right)/(BSA_i^{Mode\ 1} + 1)$, where $Diff_{rel}(BSA_i)$ denotes the relative difference of a BSA estimate of structure *i* between both modes. Box plots were drawn as in Figure S5.

Table S25. Comparison of BSA without and with the requirement that atoms are solvent exposed, relative difference per structure.

Label	N	Quartile 1	Median	Quartile 3	Mean	Std. dev.
Protein	290	7.801	9.058	11.499	10.173	3.980
Protein backbone	290	12.609	16.904	24.901	21.210	13.225
Protein side chain	290	5.833	7.054	9.143	8.090	3.475
Ligand	290	3.361	3.751	4.063	3.701	0.507
Ligand polar	290	2.125	2.766	3.658	3.068	1.472
Ligand hydrophobic	290	3.752	4.128	4.642	4.245	0.824

Values were calculated as $Diff_{rel}(BSA_i) = \left((BSA_i^{Mode\ 4} + 1) - (BSA_i^{Mode\ 1} + 1)\right)/(BSA_i^{Mode\ 1} + 1)$, where $Diff_{rel}(BSA_i)$ denotes the relative difference of a BSA estimate of structure *i* between both modes. N is the total number of differences and std. dev. refers to the standard deviation. The protein-ligand dataset was employed.

3 Acknowledgements

We thank the authors of DNAproDB (J. M. Sagendorf, H. M. Berman, and R. Rohs) for kindly calculating the DNAproDB analysis for our protein-DNA dataset. We also thank Dr. Tomás Norambuena for his box plot program.

4 References

Altschul,S.F. *et al.* (1990) Basic local alignment search tool. *J. Mol. Biol.*

Cecka,C. *et al.* (2007) Thompson Applet.

Chothia,C. (1975) Structural invariants in protein folding. *Nature*, **254**, 304–308.

Fraczkiewicz,R. and Braun,W. (1998) Exact and efficient analytical calculation of the accessible surface areas and their gradients for macromolecules. *J. Comput. Chem.*

Hubbard,S.J. and Thornton,J.M. (1993) NACCESS Department of Biochemistry and Molecular Biology, University College London.

Krissinel,E. and Henrick,K. (2007) Inference of Macromolecular Assemblies from Crystalline State. *J. Mol. Biol.*

Lide,D.R. (2001) CRC handbook of chemistry and physics CRC Press.

Liu,Z. *et al.* (2017) Forging the Basis for Developing Protein-Ligand Interaction Scoring Functions. *Acc. Chem. Res.*, **50**, 302–309.

Mitternacht,S. (2016) FreeSASA: An open source C library for solvent accessible surface area calculations. *F1000Research*, **5**, 189.

Norambuena,T. and Melo,F. (2010) The Protein-DNA Interface database. *BMC Bioinformatics*, **11**, 262.

Pettersen,E.F. *et al.* (2004) UCSF Chimera - A visualization system for exploratory research and analysis. *J. Comput. Chem.*

Ribeiro,J. *et al.* (2015) PDIviz: Analysis and visualization of protein-DNA binding interfaces. *Bioinformatics*, **31**, 2751–2753.

Saff,E.B. and Kuijlaars,A.B.J. (1997) Distributing many points on a sphere. *Math. Intell.*

Sagendorf,J.M. *et al.* (2017) DNAproDB: An interactive tool for structural analysis of DNA-protein complexes. *Nucleic Acids Res.*

Sanner,M.F. *et al.* (1996) Reduced surface: An efficient way to compute molecular surfaces. *Biopolymers*, **38**, 305–320.

Shrake,A. and Rupley,J.A. (1973) Environment and exposure to solvent of protein atoms. Lysozyme and insulin. *J. Mol. Biol.*, **79**, 351–364.

The PyMOL Molecular Graphics System (2018) Schrödinger, LLC.

Touw,W.G. *et al.* (2015) A series of PDB-related databanks for everyday needs. *Nucleic Acids Res.*

Tsai,J. *et al.* (1999) The packing density in proteins: standard radii and volumes. *J. Mol. Biol.*, **290**, 253–266.

Winn,M.D. *et al.* (2011) Overview of the CCP4 suite and current developments. *Acta Crystallogr. Sect. D Biol. Crystallogr.*
